# Lightweight Visual Detection Framework for Accurate Pepper Pest Detection Under Complex Field Conditions

**DOI:** 10.3390/insects17080822

**Published:** 2026-08-07

**Authors:** Min Dai, Chiyu Hu, Hong Miao

**Affiliations:** 1 College of Mechanical Engineering, Yangzhou University, Yangzhou 225127, China; 2 Vegetable (Root Vegetable) Fully Mechanized Research Base, Ministry of Agriculture and Rural Affairs, Yangzhou 225127, China

**Keywords:** pest management, small object detection, feature fusion, precision agriculture

## Abstract

Early pest infestations can significantly reduce chili pepper yield and quality. Therefore, timely and accurate pest detection is essential for precision pest management. However, pest detection under practical cultivation conditions remains challenging because of small target sizes, dense distributions, partial occlusion, and complex background interference. To address these challenges, this study proposes HCFD-YOLOv8, a task-oriented lightweight visual detection framework based on YOLOv8n for pepper pest monitoring in complex agricultural environments. A novel Cross-Stage Partial Hybrid Spatial Attention (CSP-HSA) module is developed as the core feature enhancement component. It is designed to improve fine-grained pest feature extraction while reducing computational redundancy. CSP-HSA combines cross-stage partial feature reuse, heterogeneous convolution, lightweight spatial-channel attention, and channel shuffle to enhance discriminative representations of small and densely distributed pests. In addition, a Cross-Scale Context Fusion Module (CCFM) is introduced to improve information interaction between high-resolution spatial details and high-level semantic features across different scales. DyHead and Inner-MPDIoU are further incorporated as complementary components to enhance adaptive feature perception and bounding-box localization, especially for partially occluded and overlapping targets. Experimental results show that YOLOv8n achieves a Precision of 84.0% and an mAP50 of 87.1%, whereas HCFD-YOLOv8 improves these values to 90.9% and 92.0%, respectively, corresponding to increases of 6.9 and 4.9 percentage points. Meanwhile, the proposed framework reduces the number of parameters, model weight-file size, and FLOPs from 3.00 M to 2.43 M, from 5.97 MB to 4.96 MB, and from 7.5 G to 6.5 G, respectively. The model achieves an inference throughput of 39.40 FPS on the evaluated cloud-server platform. Comprehensive ablation experiments demonstrate that DyHead delivers the largest standalone improvement in detection accuracy, whereas CSP-HSA provides a more favorable trade-off between feature enhancement and computational efficiency. CCFM and Inner-MPDIoU further provide complementary improvements in cross-scale feature representation and bounding-box localization. These results indicate that HCFD-YOLOv8 achieves a favorable balance between detection accuracy and computational efficiency, demonstrating its potential for lightweight and accurate pepper pest monitoring under complex agricultural conditions.

## Simple Summary

Chili pepper production is seriously threatened by insect pests, especially small pests such as aphids, thrips, and caterpillars. Early and accurate detection of these pests is essential for effective pest management. However, pest detection under practical cultivation conditions remains challenging. The main reasons are the small size of pest targets, dense aggregation, partial occlusion by leaves, and interference from complex plant backgrounds. These factors make manual inspection inefficient and prone to errors. To overcome these limitations, this study develops an artificial intelligence-based visual detection method for automatic pepper pest identification in open-field and greenhouse environments. The proposed lightweight model enhances pest feature extraction, improves multi-scale feature interaction, and increases localization accuracy for small, dense, and partially occluded targets. Experimental results demonstrate that the proposed method achieves higher detection accuracy than the baseline model while reducing model parameters, computational cost, and weight-file size. Moreover, it maintains efficient inference performance on the evaluated cloud-server platform. These results indicate that the proposed method has potential for accurate and efficient pepper pest monitoring. Further validation on embedded devices and under more diverse field conditions is still required.

## 1. Introduction

Pepper (*Capsicum annuum* L.) is one of the most important vegetable and economic crops worldwide. It is widely cultivated for fresh consumption, food processing, and condiment production [1]. According to recent FAOSTAT statistics, global pepper production exceeds 37 million tonnes annually. However, pepper production is frequently threatened by insect pests, including aphids, thrips, whiteflies, and caterpillars. These pests can directly damage plant tissues and transmit plant pathogens, causing substantial yield and quality losses. Globally, crop pests and diseases are estimated to cause approximately 20–40% of annual agricultural production losses [2]. Therefore, accurate and timely pest monitoring is essential for improving crop productivity, reducing unnecessary pesticide application, and promoting sustainable agricultural development.

Traditional pest monitoring relies primarily on manual scouting, sticky traps, pheromone traps, and expert identification. Although these approaches are widely used in agricultural practice, they are labor-intensive, time-consuming, and highly dependent on expert experience. Early machine-vision methods attempted to identify pests using handcrafted features, such as color, texture, and shape descriptors, combined with traditional machine-learning algorithms. However, these methods often exhibit limited robustness under practical field conditions. Variations in illumination, complex backgrounds, target occlusion, and pest morphological diversity can significantly affect detection performance.

The rapid development of deep learning has greatly advanced agricultural image analysis and intelligent pest monitoring. Since Krizhevsky et al. introduced AlexNet [3], deep convolutional neural networks (CNNs) have demonstrated strong feature-learning capabilities and have become fundamental techniques in visual recognition. Subsequently, deep learning-based object detection methods have been widely applied in agricultural pest monitoring. Current object detection frameworks are mainly categorized into two-stage and one-stage detectors.

Two-stage detectors, such as Faster R-CNN [4], AF-RCNN [5], CRA-Net [6], and Cascade R-CNN [7], generally achieve high localization accuracy by generating region proposals before classification and regression. For example, Wang et al. developed AF-RCNN to improve pest proposal generation and localization performance in agricultural scenes. Dong et al. proposed CRA-Net [8] by introducing a Channel Recalibration Feature Pyramid Network (CRFPN) and an Adaptive Anchor module to enhance small pest detection. However, the complex architectures and high computational costs of these methods limit their application in real-time agricultural monitoring.

Compared with two-stage detectors, one-stage detectors provide faster inference and simpler deployment. Among them, the YOLO family has attracted considerable attention because of its balance between detection accuracy and computational efficiency. Bochkovskiy et al. proposed YOLOv4 [9], which integrates CSPDarknet53, Spatial Pyramid Pooling (SPP), PANet feature aggregation, Mosaic augmentation, and CIoU loss to achieve accurate and efficient object detection. Due to their practical efficiency, YOLO-based frameworks have become widely used in agricultural pest detection.

Many YOLO-based studies have attempted to improve pest detection through enhanced feature extraction and multi-scale representation. For small orchard pest detection, Tian et al. proposed MD-YOLO [10] by introducing dense feature reuse and multi-scale feature extraction strategies. Tang et al. developed Improved Pest-YOLO [11] by incorporating Efficient Channel Attention (ECA), Transformer Encoder, and Cross-Stage Feature Fusion (CSFF), achieving improved performance on the Pest24 dataset. Zheng et al. proposed Paddy-YOLO [12] with an RGG feature extraction module and IMPDIoU loss, achieving a 9.1% improvement in mAP50. Wang et al. developed CBF-YOLO [13] to enhance cross-scale feature interaction for soybean pest detection. Zhang et al. proposed CPD-YOLO [14] by integrating BiFPN, Vision Transformer, and Dynamic Head structures for improved feature aggregation under complex field conditions.

Attention mechanisms have become an important strategy for improving feature representation in agricultural pest detection. By adaptively enhancing informative regions and suppressing irrelevant responses, attention-based methods can improve the detection of small and visually similar pests under complex environments. Ma et al. proposed MH-YOLO [15] by integrating hybrid attention strategies and transfer learning to enhance orchard pest recognition. Hou et al. developed RS-YOLO [16], which introduced EMA attention to strengthen detailed feature extraction and reduce confusion among visually similar pest categories. Similar attention-guided strategies have also been explored in other agricultural vision tasks, such as PP-YOLO [17] and YOLO-detassel [18], demonstrating the potential of attention mechanisms for improving feature discrimination.

In addition to feature enhancement, recent studies have addressed long-tail distributions and lightweight deployment. Chen Zhang et al. proposed LTF-YOLO [19] with a Pest Neighborhood Attention Transformer (PNAT), downsampling, and Balanced Proxy Loss (BP Loss), achieving 77.7% mAP50 on the MCP15 dataset. Li et al. proposed Generalized Focal Loss (GFL) [20] to jointly optimize classification confidence and localization uncertainty. Yu Li et al. introduced Balanced Group Softmax (BAGS) [21] to mitigate classifier bias caused by long-tail distributions. Lightweight architectures such as YOLO-MRC [22] and RepViT [23] further demonstrate that efficient models can maintain competitive detection performance while reducing computational complexity.

Although these studies have significantly advanced agricultural pest detection, several limitations remain. First, most existing studies focus on rice, soybean, maize, cotton, or mixed-crop datasets, while dedicated datasets and detection frameworks for pepper pests are still limited. Second, pepper pests usually exhibit small target sizes, dense distributions, partial occlusion, and strong background interference, which makes fine-grained feature extraction difficult. Third, many existing methods achieve higher accuracy by increasing network complexity, resulting in higher computational costs and limiting their application in lightweight agricultural monitoring systems. Therefore, developing a lightweight detection framework that can simultaneously improve feature extraction, maintain multi-scale representation capability, and achieve accurate localization remains an important research challenge for pepper pest monitoring.

Recent studies have extended vision-based insect monitoring from offline image analysis toward automated and field-oriented monitoring systems. Santaera et al. [24] developed an autonomous smart trap for precision monitoring of hematophagous flies on cattle. Their work demonstrated the feasibility of integrating image acquisition, insect recognition, and autonomous monitoring into practical sensing systems. Rajeswaran et al. [25] evaluated computationally efficient deep learning models for live insect classification and detection, highlighting the importance of balancing detection accuracy and computational cost. These studies provide valuable insights into the application of computer vision for automated insect monitoring. However, their research objectives and application scenarios differ from pepper pest detection. Santaera et al. mainly focused on trap-based monitoring of livestock-associated flies, while Rajeswaran et al. evaluated general-purpose insect detection models. These studies did not specifically consider the combined challenges encountered in pepper fields, including extremely small targets, dense pest aggregation, leaf occlusion, and complex plant backgrounds.

More recently, Shi et al. [26] proposed RSA-YOLO, an improved YOLO11-based framework for chili pepper pest and disease detection. By introducing a Super Convolutional Block Attention Module (SPCBAM) and a Reparameterized Heterogeneous Multiscale Structure (RepHMS), RSA-YOLO improved feature representation and reduced background interference in complex agricultural scenes. The model achieved an average precision of 80.5%, representing an 8% improvement over the original YOLO11 while maintaining a lightweight structure. This study demonstrates the effectiveness of YOLO-based optimization strategies for chili pepper-related visual detection. However, RSA-YOLO mainly focuses on joint pest and disease recognition using public benchmark datasets. Its improvements primarily target backbone feature enhancement through RepHMS and SPCBAM, while the coordinated optimization of feature extraction, multi-scale information interaction, adaptive prediction, and localization accuracy for naturally occurring pepper pests remains insufficiently explored.

Compared with existing YOLO-based agricultural detection methods, the present study focuses on pepper pests collected from open-field and greenhouse environments. These practical scenarios involve multiple challenges, including small target sizes, dense distributions, target overlap, partial occlusion, illumination variation, and complex leaf backgrounds. Such factors weaken fine-grained feature extraction, hinder effective multi-scale feature interaction, and reduce localization accuracy.

Despite significant advances in agricultural object detection, several challenges remain unresolved for practical pepper pest monitoring. First, many existing pest detection studies mainly focus on laboratory datasets, trap-based monitoring scenarios, or mixed-crop pest recognition tasks. Although these studies demonstrate the feasibility of deep learning-based insect monitoring, their application scenarios differ substantially from natural pepper cultivation environments, where pests usually appear as extremely small targets with irregular distribution, dense aggregation, partial occlusion, and strong interference from leaf textures and illumination variations. Second, current accuracy-oriented detection methods often improve performance by introducing complex attention mechanisms, Transformer structures, or additional feature fusion pathways. While these strategies enhance feature representation, they inevitably increase computational cost and model complexity, which limits their application in lightweight agricultural monitoring systems. Third, existing approaches generally optimize individual components of object detection frameworks separately. For example, some studies focus on backbone feature enhancement, whereas others improve multi-scale fusion, detection heads, or localization losses. However, a coordinated optimization strategy that simultaneously considers feature extraction, cross-scale interaction, adaptive prediction, and localization accuracy for small and densely distributed pepper pests remains insufficiently explored.

To address these limitations, this study proposes HCFD-YOLOv8, a task-oriented lightweight detection framework specifically designed for pepper pest monitoring under complex agricultural environments. Instead of simply combining existing modules, the proposed framework follows a hierarchical optimization principle according to different stages of the detection pipeline. Specifically, CSP-HSA is designed as a lightweight feature enhancement module in the backbone network to improve fine-grained pest representation while reducing redundant computation. CCFM is introduced into the neck network to strengthen cross-scale information exchange between detailed spatial features and high-level semantic information. DyHead is incorporated into the detection head to enhance adaptive perception under scale variation and background interference. Inner-MPDIoU is employed during regression optimization to improve localization accuracy for overlapping and partially occluded targets.

Unlike a simple combination of existing modules, HCFD-YOLOv8 establishes a coordinated optimization framework in which different components address different limitations of pepper pest detection. The main novelty of this study lies in the proposed CSP-HSA module and its integration into a lightweight, task-specific detection framework. By combining efficient feature extraction, cross-scale feature interaction, adaptive prediction, and accurate localization, HCFD-YOLOv8 aims to achieve a better balance between detection accuracy and computational efficiency under complex pepper cultivation conditions. Finally, to improve the readability of the paper, we list the descriptions of terminologies, abbreviations, and mathematical symbols in Appendix A.

The contributions of this study are summarized as follows:

(1) A task-oriented lightweight detection framework, termed HCFD-YOLOv8, is proposed for small and densely distributed pepper pests under complex open-field and greenhouse conditions. Unlike approaches that improve accuracy by continuously increasing model complexity, the proposed framework adopts a hierarchical optimization strategy to jointly enhance feature extraction, multi-scale feature interaction, adaptive prediction, and bounding-box localization, achieving a better balance between detection performance and computational efficiency.
(2) A novel Cross-Stage Partial Hybrid Spatial Attention (CSP-HSA) module is proposed as the core feature enhancement component. The module integrates cross-stage partial feature reuse, heterogeneous convolution, lightweight spatial-channel attention, and channel shuffle to enhance fine-grained pest feature extraction while reducing redundant computation. In addition, a Cross-Scale Context Fusion Module (CCFM) is introduced to improve the interaction between high-resolution spatial details and high-level semantic information for pests with different scales.
(3) DyHead and Inner-MPDIoU are incorporated as complementary optimization components for adaptive detection and accurate localization. DyHead dynamically refines multi-level features before prediction to improve target perception under scale variation and background interference, while Inner-MPDIoU strengthens geometric constraints during bounding-box regression. Their individual contributions and combined effects are systematically evaluated through ablation and comparative experiments.
(4) The proposed framework is evaluated on a pepper pest dataset collected under realistic agricultural conditions, including small targets, dense aggregation, partial occlusion, target overlap, and complex backgrounds. Experimental results demonstrate that HCFD-YOLOv8 improves detection accuracy while reducing parameter count and computational cost compared with the YOLOv8n baseline, indicating its potential for efficient pepper pest monitoring in complex cultivation environments.

## 2. Materials and Methods

### 2.1. Overview

**Figure 1:**
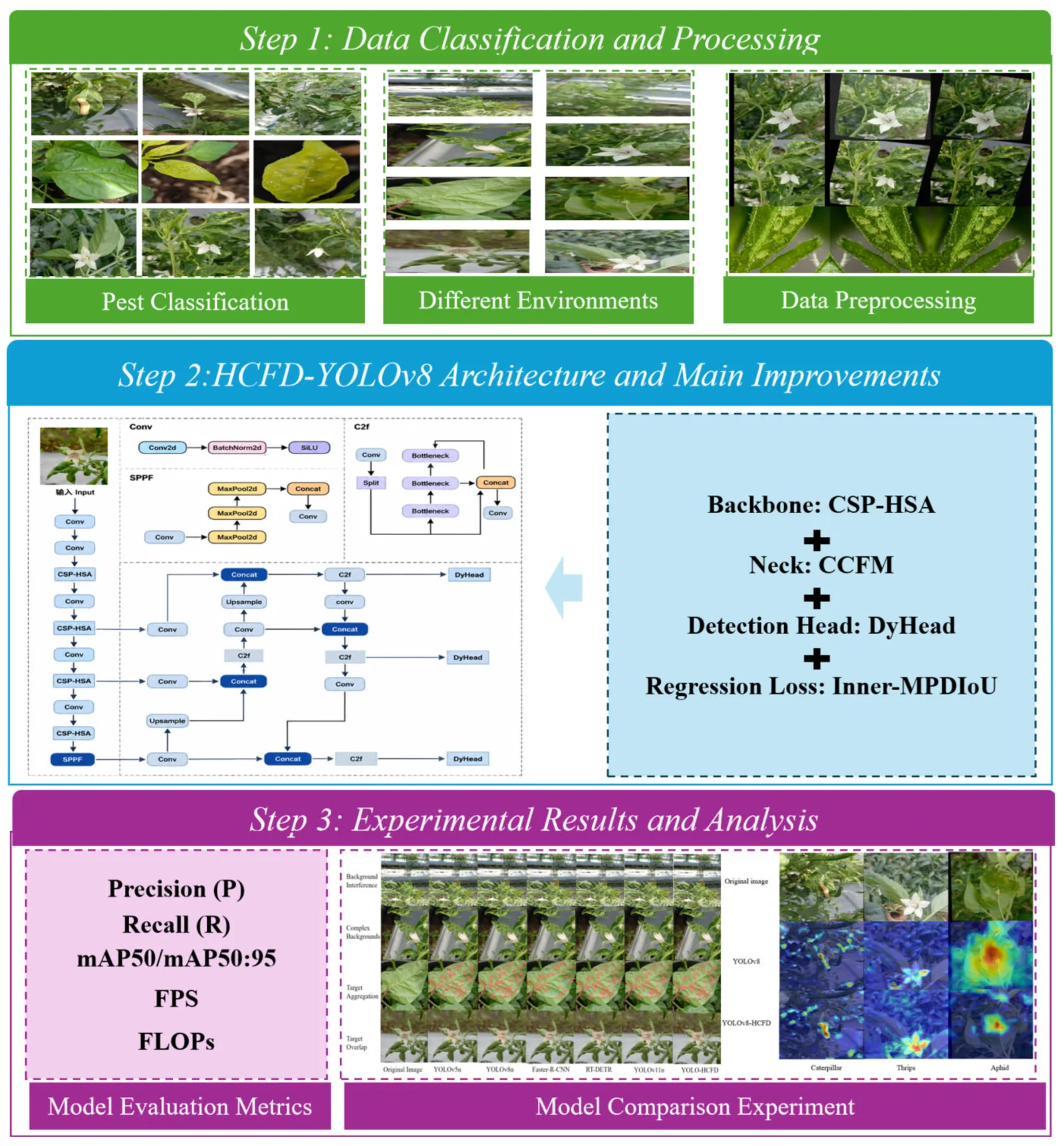
Overall workflow of the proposed HCFD-YOLOv8 framework, including dataset construction and preprocessing, network construction and optimization, and experimental evaluation. In Step 2, the overall network architecture and four principal modifications to YOLOv8n are illustrated, including CSP-HSA in the backbone, CCFM in the neck, DyHead in the detection head, and Inner-MPDIoU for bounding-box regression.

This study proposes a lightweight object detection framework, HCFD-YOLOv8, for accurate chili pepper pest detection under complex agricultural conditions. The overall workflow of the proposed framework is illustrated in Figure 1.

Step 1: Dataset construction and preprocessing. A chili pepper pest dataset was constructed using images collected from field environments and publicly available datasets. The dataset includes three representative pest categories: caterpillars, aphids, and thrips. To improve model robustness under practical conditions, the collected images contain diverse scenarios, including background interference, complex vegetation environments, target aggregation, and target overlap. The dataset was then processed through annotation, format conversion, quality filtering, and normalization. Data augmentation strategies, including flipping, rotation, noise injection, and Mosaic augmentation, were subsequently applied to increase data diversity.

Step 2: Model training and optimization. The processed dataset was used to train and optimize the proposed HCFD-YOLOv8 framework. In the backbone network, the original C2f module was replaced with the proposed CSP-HSA module to enhance fine-grained feature extraction for small and densely distributed pests while reducing redundant feature responses. In the neck network, the CCFM module was introduced to improve cross-scale feature interaction and strengthen multi-scale representation. In the detection head, DyHead was incorporated to enable adaptive feature refinement across spatial and semantic dimensions. Finally, the original CIoU loss was replaced with Inner-MPDIoU to improve bounding-box regression by introducing stronger geometric constraints.

Step 3: Model evaluation and analysis. The performance of the proposed framework was evaluated through ablation experiments, comparative experiments, and visualization analysis. Ablation studies were conducted to quantify the contribution of each component and verify their complementary effects. Comparative experiments with representative detectors, including YOLOv5n, YOLOv8n, YOLOv11n, Faster R-CNN, and RT-DETR, were performed to evaluate detection accuracy and computational efficiency. Visualization results were further analyzed to examine the model’s attention to pest regions and its ability to reduce missed detections under complex agricultural conditions.

### 2.2. Data Acquisition

#### 2.2.1. Dataset Collection

**Figure 2:**
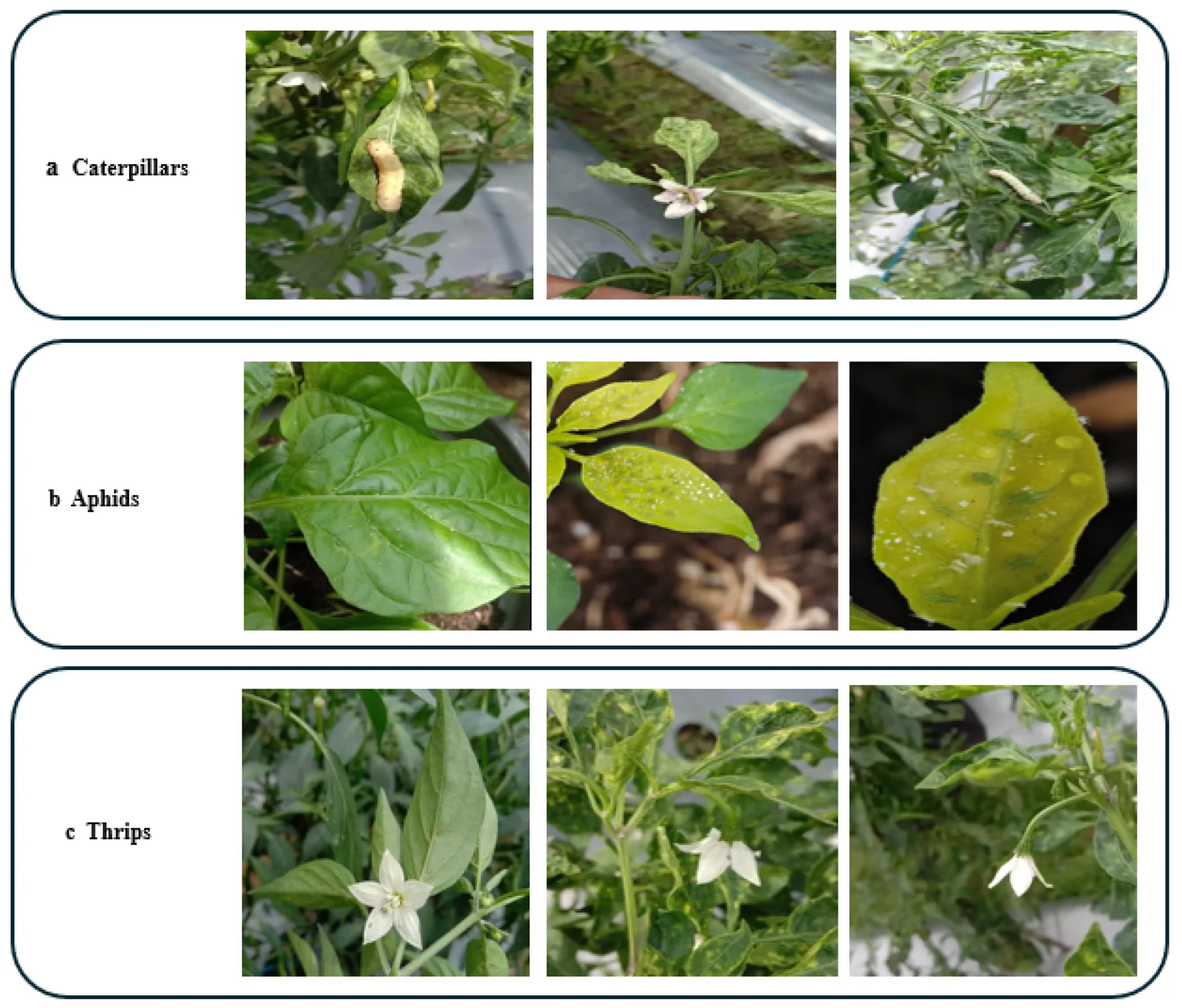
Dataset classification of three representative pests, including (**a**) caterpillars, (**b**) aphids, and (**c**) thrips.

The image dataset used in this study was collected from two sources: images collected under pepper cultivation environments and publicly available agricultural image repositories. The field and greenhouse images were collected from pepper cultivar Bo 15 (*Capsicum annuum* L.) using a vivo X300 smartphone (Vivo Mobile Communication Co., LTD., Dongguan, China) at the Yangzhou Agricultural Science and Technology Center and the greenhouse of Yangzhou University, respectively. The open-field plants were grown under natural environmental conditions, while the greenhouse plants were maintained under controlled protected cultivation conditions. Both cultivation systems followed standard agricultural management practices, including regular irrigation, fertilization, and routine crop maintenance. During image acquisition, no artificial modifications were applied to the plants or pest populations. Therefore, the collected images reflected natural pest occurrence conditions during crop growth. Additional images were obtained from publicly available agricultural image repositories, including the Baidu AI Studio dataset platform (https://aistudio.baidu.com/datasetdetail/153190, accessed on 18 May 2026). These third-party images were used only for academic research purposes and were not redistributed due to copyright restrictions. Based on these image sources, a chili pepper pest dataset was established. As shown in Figure 2, the dataset contains three representative pest categories: caterpillars, aphids, and thrips. Detailed dataset information and annotation files generated in this study are available from the corresponding author upon reasonable request.

To better represent practical agricultural environments, the collected images included various visual conditions that may affect pest detection performance. These conditions included illumination changes caused by different acquisition times, complex vegetation backgrounds, variations in leaf color and morphology during crop growth, differences in pest scales, uneven pest distributions, target aggregation, partial occlusion, and overlap between pests and leaves. Although the dataset was not specifically designed for year-round seasonal monitoring, images collected under diverse plant growth conditions and environmental backgrounds were included to increase visual diversity and provide a more comprehensive evaluation of model robustness.

**Figure 3:**
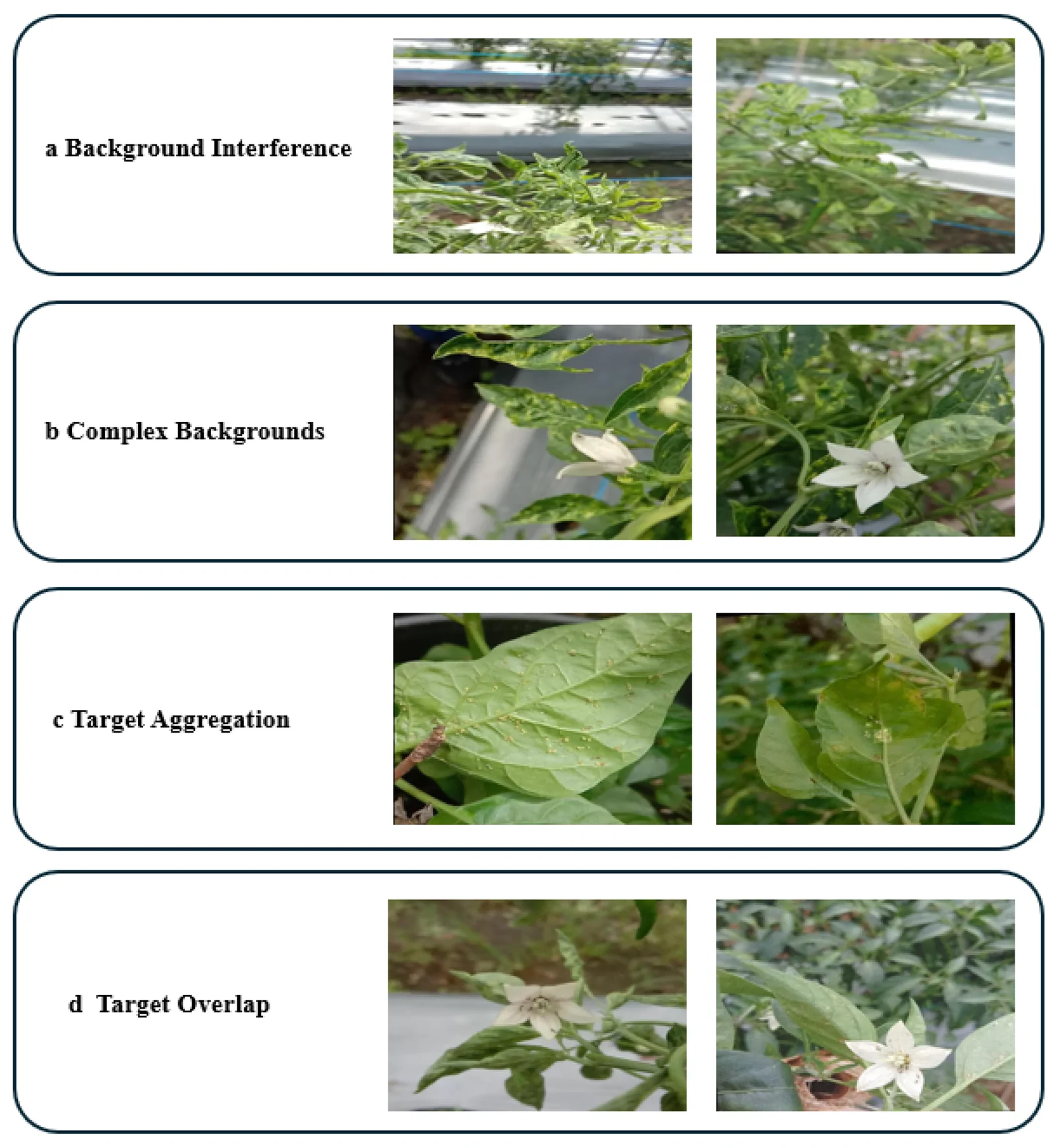
Representative images of pests under different environmental conditions. It includes (**a**) background interference, (**b**) complex backgrounds, (**c**) target aggregation, and (**d**) target overlap.

To better reflect practical agricultural conditions, pest images containing four challenging scenarios were included in the dataset. These scenarios included background interference, complex vegetation backgrounds, target aggregation, and target overlap. These diverse conditions were considered during model evaluation to improve dataset diversity and assess the robustness of the proposed framework under complex agricultural environments. Representative examples of these scenarios are shown in Figure 3.

#### 2.2.2. Data Augmentation

After data filtering, a total of 1035 valid original images were retained for dataset construction. Before data augmentation, the original images were randomly divided into a training set containing 535 images, a validation set containing 250 images, and a testing set containing 250 images. Relatively large validation and testing subsets were retained to provide reliable model selection and independent performance evaluation under diverse agricultural conditions. Data augmentation was applied only to the training set, including Mosaic augmentation, random translation, horizontal and vertical flipping, rotation, and Gaussian noise injection.

After augmentation, the number of training images increased from 535 to 2005, resulting in a final dataset containing 2505 images. The validation and testing sets remained unchanged and were used for model selection and final performance evaluation, respectively. The dataset distribution after augmentation is presented in Table 1. Although augmentation increases the number of training samples, it does not generate new biological diversity. Therefore, the current dataset should be considered a representative dataset for evaluating model performance under the included cultivation conditions rather than a comprehensive representation of all pepper production environments.

### 2.3. Novel Network Construction

Pepper pest detection under practical agricultural conditions faces several challenges, including small object size, complex backgrounds, dense distributions, target overlap, and localization uncertainty caused by irregular pest boundaries. These challenges occur at different stages of the object detection pipeline and are difficult to fully address using a single optimization strategy.

To address these diverse challenges, this study proposes a hierarchical collaborative optimization strategy within the HCFD-YOLOv8 framework, as shown in Table 2. Specifically, the proposed CSP-HSA module is integrated into the backbone network to enhance low-level feature extraction through spatial–channel feature interaction and adaptive feature recalibration. In the neck network, CCFM is introduced to strengthen cross-scale feature fusion by facilitating information exchange between high-resolution spatial details and high-level semantic representations. In the detection head, DyHead is incorporated to adaptively refine feature responses according to object scales, spatial distributions, and prediction requirements. Finally, Inner-MPDIoU is employed as the regression optimization strategy to improve localization accuracy by strengthening geometric constraints between predicted and ground-truth bounding boxes. Since these components are designed for different stages of the detection pipeline, they provide complementary improvements rather than redundant feature enhancement. Through progressive optimization of feature extraction, multi-scale representation, adaptive prediction, and bounding-box regression, HCFD-YOLOv8 improves the detection capability of small and densely distributed pepper pests under complex agricultural environments.

To select an appropriate baseline model for chili pepper pest detection, three representative lightweight object detectors, including YOLOv5n, YOLOv8n, and YOLOv11n, were evaluated under identical dataset and training conditions. The comparison results are summarized in Table 3.

After comparing different YOLO series, YOLOv8n is considered in this study. Although YOLOv11n achieved lower parameter numbers and FLOPs, its detection accuracy was slightly inferior to that of YOLOv8n on the chili pepper pest dataset. Specifically, YOLOv8n achieved an mAP50 of 87.1% and an mAP50:95 of 54.3%, which were 0.6 percentage points higher than those of YOLOv11n, respectively. This performance difference is important for pepper pest detection because the dataset contains challenging characteristics, including small targets, dense pest distributions, partial occlusion, and complex backgrounds. Under such conditions, maintaining detection accuracy is essential for reliable pest recognition. Furthermore, YOLOv8n provides a stable and widely validated architecture with favorable modular compatibility, which facilitates the integration of the proposed CSP-HSA, CCFM, DyHead, and Inner-MPDIoU components. Therefore, considering detection accuracy, model stability, architectural adaptability, and computational efficiency, YOLOv8n was selected as the baseline framework for subsequent optimization.

The YOLOv8n architecture consists of four main components: the input processing stage, backbone network, neck network, and detection head. In the input stage, YOLOv8n employs data augmentation strategies, adaptive label assignment, and image preprocessing techniques to improve data diversity and training stability.

The backbone network adopts a feature extraction structure composed of convolutional layers, C2f modules, and the Spatial Pyramid Pooling Fast (SPPF) module. The C2f module is derived from the ELAN concept introduced in YOLOv7 and enhances feature propagation through partial feature reuse and cross-stage connections, thereby improving representation capability while maintaining computational efficiency. The SPPF module expands the receptive field through multi-scale pooling operations and strengthens contextual feature extraction.

The neck network adopts a Path Aggregation Network (PANet)-based feature fusion strategy to integrate information from different scales. By combining low-level spatial details with high-level semantic representations, the neck improves the perception ability of objects with varying sizes. The detection head adopts an anchor-free decoupled structure, where classification and bounding-box regression tasks are optimized separately. This design reduces task interference and improves prediction efficiency.

Although YOLOv8n provides an effective balance between accuracy and computational cost, its performance remains limited in complex agricultural environments, particularly for small objects, large appearance variations, partial occlusion, and strong background interference. To address these challenges, this study proposes HCFD-YOLOv8, a lightweight chili pepper pest detection framework based on YOLOv8n. The proposed framework introduces targeted improvements in feature extraction, multi-scale feature interaction, adaptive prediction, and localization regression.

#### 2.3.1. CSP-HSA Module

To overcome the limitations of the original C2f module in extracting discriminative features from small and densely distributed pepper pests, a lightweight Cross-Stage Partial Hybrid Spatial Attention (CSP-HSA) module is proposed and integrated into the YOLOv8 backbone.

The proposed CSP-HSA module is designed based on the Cross Stage Partial (CSP) principle and consists of two parallel pathways: a feature transformation branch and a shortcut branch. This design enables partial feature reuse and efficient gradient propagation while reducing unnecessary feature redundancy. Compared with the original C2f module, CSP-HSA introduces adaptive feature refinement mechanisms to enhance the representation of pest-related information under complex agricultural backgrounds.

The feature transformation branch incorporates a Hybrid Spatial Attention (HSA) unit to enhance discriminative feature learning. Unlike standard feature extraction, which mainly relies on local convolution responses, the HSA unit jointly models channel relationships and spatial contextual information. Through adaptive attention weighting, the HSA unit recalibrates the input features across the channel and spatial dimensions. This design is intended to strengthen the representation of informative pest features, particularly for small targets with weak visual characteristics.

To maintain computational efficiency, lightweight convolution operations are adopted to reduce redundant parameter learning during feature extraction. In addition, channel shuffle is introduced to promote information exchange among channel groups and alleviate the limited cross-group interaction caused by grouped operations. These designs enable CSP-HSA to improve feature diversity without introducing substantial computational overhead.

**Figure 4:**
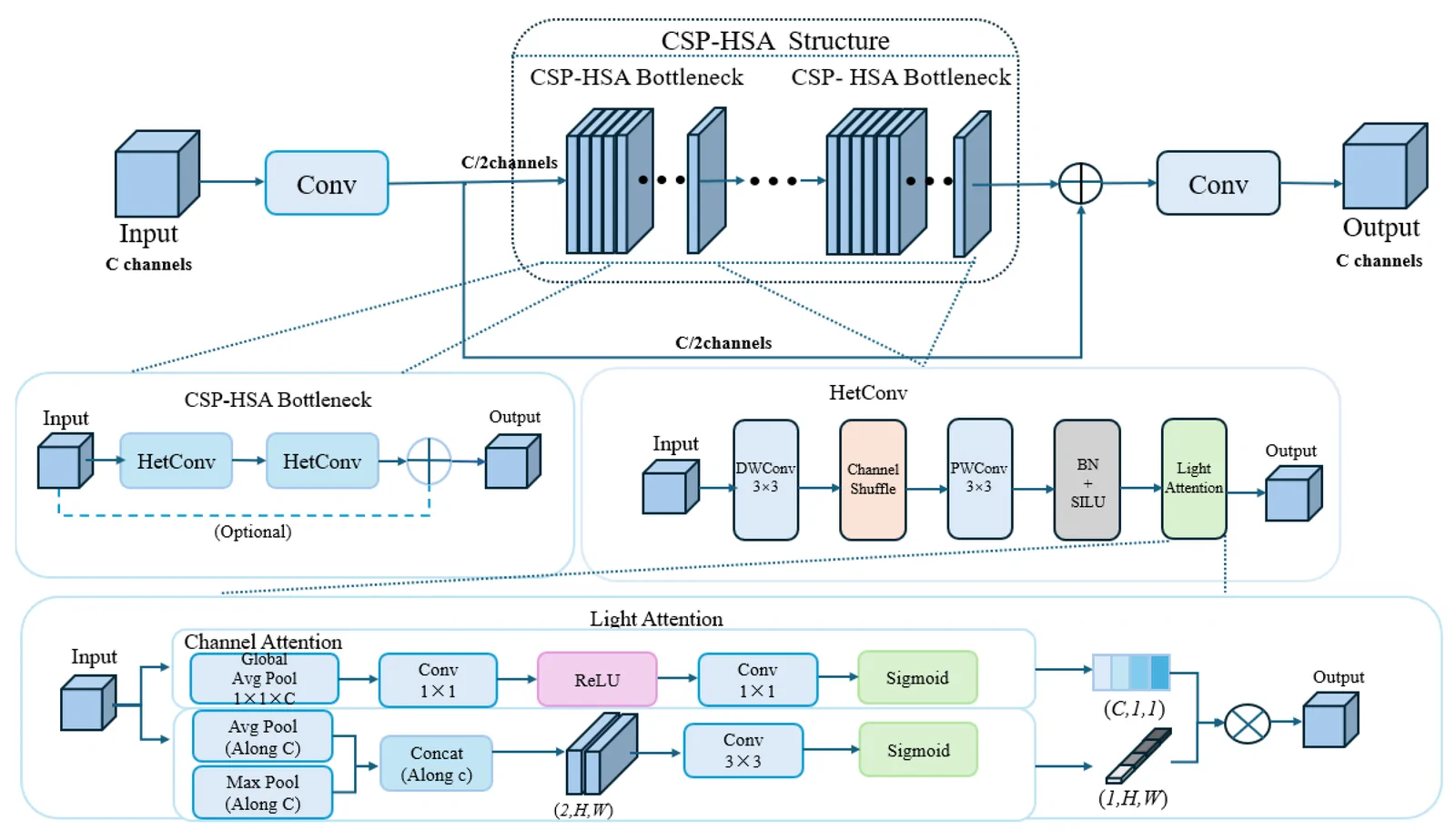
Structure of the CSP-HSA module for efficient feature extraction and attention enhancement. The module consists of CSP-HSA bottlenecks, HetConv blocks, and a Light Attention mechanism. Light Attention contains channel and spatial attention branches, which recalibrate feature responses by emphasizing pest-related channels and spatial regions while suppressing redundant background information with limited computational overhead.

By integrating CSP-based feature reuse, lightweight convolution, channel interaction enhancement, and spatial-channel attention refinement, CSP-HSA establishes a more effective feature representation mechanism for complex pepper pest detection. The module improves fine-grained feature extraction and contextual perception while maintaining a lightweight architecture, providing more discriminative representations for subsequent detection stages. The detailed architecture of CSP-HSA is illustrated in Figure 4.

The Light Attention module illustrated in Figure 4 serves as a lightweight feature recalibration component within CSP-HSA. It is designed to enhance informative pest features while maintaining low computational overhead. The module consists of two complementary branches: channel attention and spatial attention. In the channel attention branch, global average pooling and global max pooling are employed to generate channel-level feature descriptors. These descriptors are further transformed through lightweight convolutional operations and nonlinear activation functions to obtain channel-wise attention weights. The generated weights adaptively emphasize channels containing discriminative pest information. In the spatial attention branch, average pooling and max pooling are performed along the channel dimension to aggregate spatial information. The resulting feature descriptors are concatenated and processed using a convolutional layer to generate a spatial attention map. This attention map adaptively reweights spatial locations and strengthens feature responses in potentially informative regions. The channel and spatial attention weights are subsequently combined through element-wise multiplication to recalibrate the input feature representation. Compared with computationally expensive attention mechanisms, the proposed Light Attention module achieves effective feature refinement using lightweight operations, introducing limited additional parameters and computational cost.

In addition, CSP-HSA incorporates a lightweight Hybrid Spatial Attention (HSA) unit to improve feature interaction across channel and spatial dimensions. The HSA unit is specifically designed to enhance representation capability while preserving the lightweight characteristics of the framework. It integrates heterogeneous convolution, depthwise separable convolution, and channel shuffle operations. Heterogeneous convolution enables the extraction of diverse local patterns through multiple convolutional transformations, which improves the perception of pest features with different scales and appearances. Depthwise separable convolution reduces redundant computation by decoupling spatial feature extraction and channel-wise feature fusion. Meanwhile, the channel shuffle operation enhances information exchange among different channel groups and alleviates limited interaction caused by grouped operations. Hence, through the coordinated design of Light Attention and HSA, CSP-HSA improves feature recalibration and cross-dimensional information interaction while maintaining computational efficiency. This design is particularly suitable for pepper pest detection scenarios involving small targets, dense distributions, and complex backgrounds.

**Figure 5:**
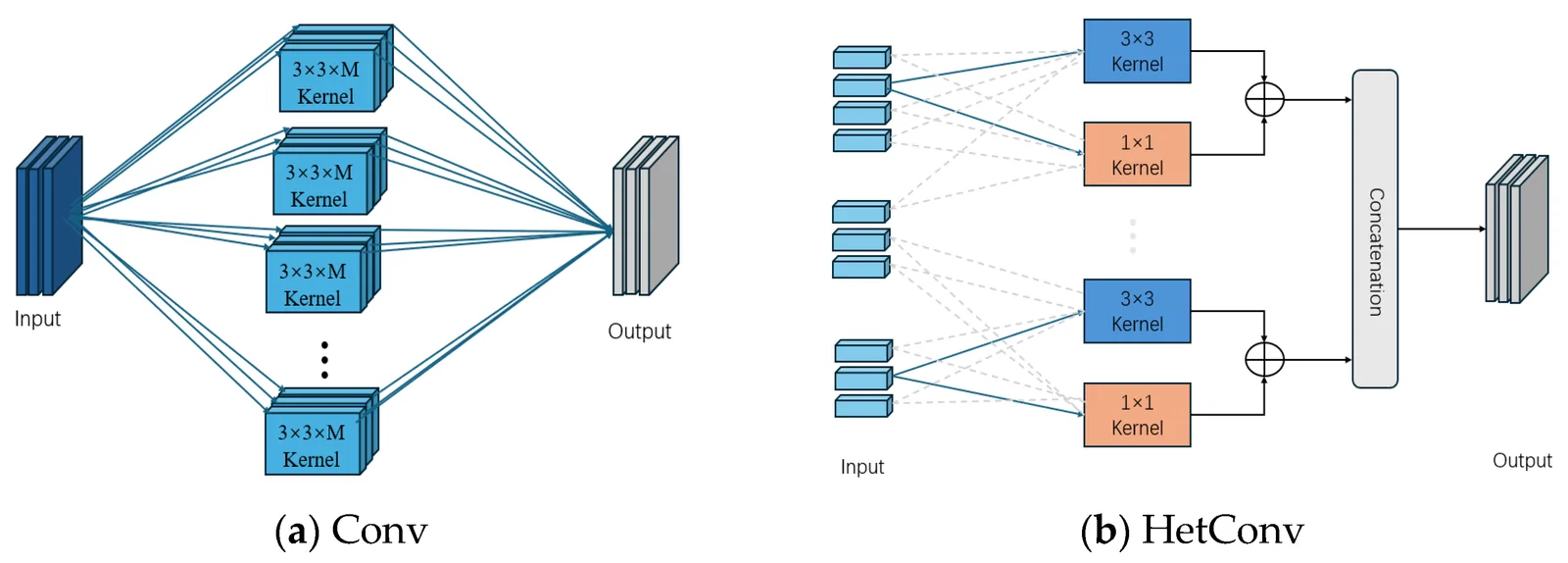
Comparison between standard convolution and HetConv. (**a**) Conv performs feature extraction using a single 3 × 3 convolution kernel. (**b**) HetConv improves efficiency by integrating heterogeneous convolution operations, combining 3 × 3 and 1 × 1 kernels for enhanced feature representation.

Furthermore, through the joint modeling of spatial and channel information, the HSA unit performs adaptive feature recalibration to strengthen discriminative feature representation. Combined with the CSP structure, CSP-HSA improves feature interaction and contextual representation while maintaining a lightweight architecture. By balancing feature enhancement and computational efficiency, the proposed module provides effective feature representations for subsequent detection stages. The lightweight architecture of the proposed CSP-HSA module is illustrated in Figure 5.

While keeping the number of parameters under control, CSP-HSA effectively enlarges the receptive field through heterogeneous convolutions. For a standard 3 × 3 convolution, the number of parameters is given by:

(1) $$P_{3\times 3}=9C_{in}C_{out}$$

where P_3×3_ is the total number of learnable parameters of a standard 3 × 3 convolution layer, and Cin and Cout denote the numbers of input and output channels, respectively. In contrast, the HetConv adopted in CSP-HSA divides the convolution kernels into Pgroups. Each group contains both 3 × 3 and 1 × 1 convolutions. Therefore, the number of parameters can be calculated as:

(2) $$P_{Het}=9(\frac{C_{in}}{P})(\frac{C_{out}}{P})+(C_{in}-\frac{C_{in}}{P})(\frac{C_{out}}{P})$$

where *C_in_* and *C_out_* denote the input and output channels, respectively. Compared with standard convolution, the heterogeneous convolution structure reduces redundant parameter calculation by replacing part of the 3 × 3 convolution operations with lightweight 1 × 1 convolutions while maintaining multi-scale feature extraction capability.

#### 2.3.2. CCFM Architecture

In the original YOLOv8 neck network, multi-scale feature fusion is mainly achieved through the combination of FPN and PAN structures. Although this design effectively aggregates features from different levels, the sequential feature aggregation process may result in insufficient preservation of spatial details and semantic consistency during cross-scale information interaction, particularly for small objects with weak visual characteristics.

To address these limitations, CCFM is introduced into the neck network to replace the original feature fusion structure. The proposed module aims to enhance multi-scale feature interaction by adaptively coordinating information exchange between high-resolution spatial features and low-resolution semantic representations.

CCFM establishes a bidirectional cross-scale interaction pathway to facilitate complementary information exchange among feature maps with different resolutions. Specifically, fine-grained spatial details from high-resolution features are integrated with semantic information from low-resolution features, enabling more effective representation of objects with different scales. During feature fusion, an adaptive feature weighting strategy is employed to adjust the contribution of different-scale features according to their relative importance. This mechanism enhances informative representations while reducing redundant responses and background interference.

Furthermore, the collaborative cross-scale design of CCFM improves feature propagation and enlarges the effective receptive field without introducing substantial computational overhead. This characteristic enhances the robustness of HCFD-YOLOv8 when detecting pepper pests with scale variation, partial occlusion, deformation, and dense distribution.

#### 2.3.3. DyHead Detection Head

In conventional YOLO-based detectors, classification and bounding-box regression are generally performed through predefined convolutional transformations in the detection head. Although this design provides efficient prediction, the fixed feature refinement process has limited adaptability when handling targets with significant scale variation, diverse appearances, and complex contextual interference. This limitation is particularly evident in pepper pest detection, where targets often exhibit small sizes, irregular shapes, partial occlusion, and large variations in visual characteristics.

**Figure 6:**
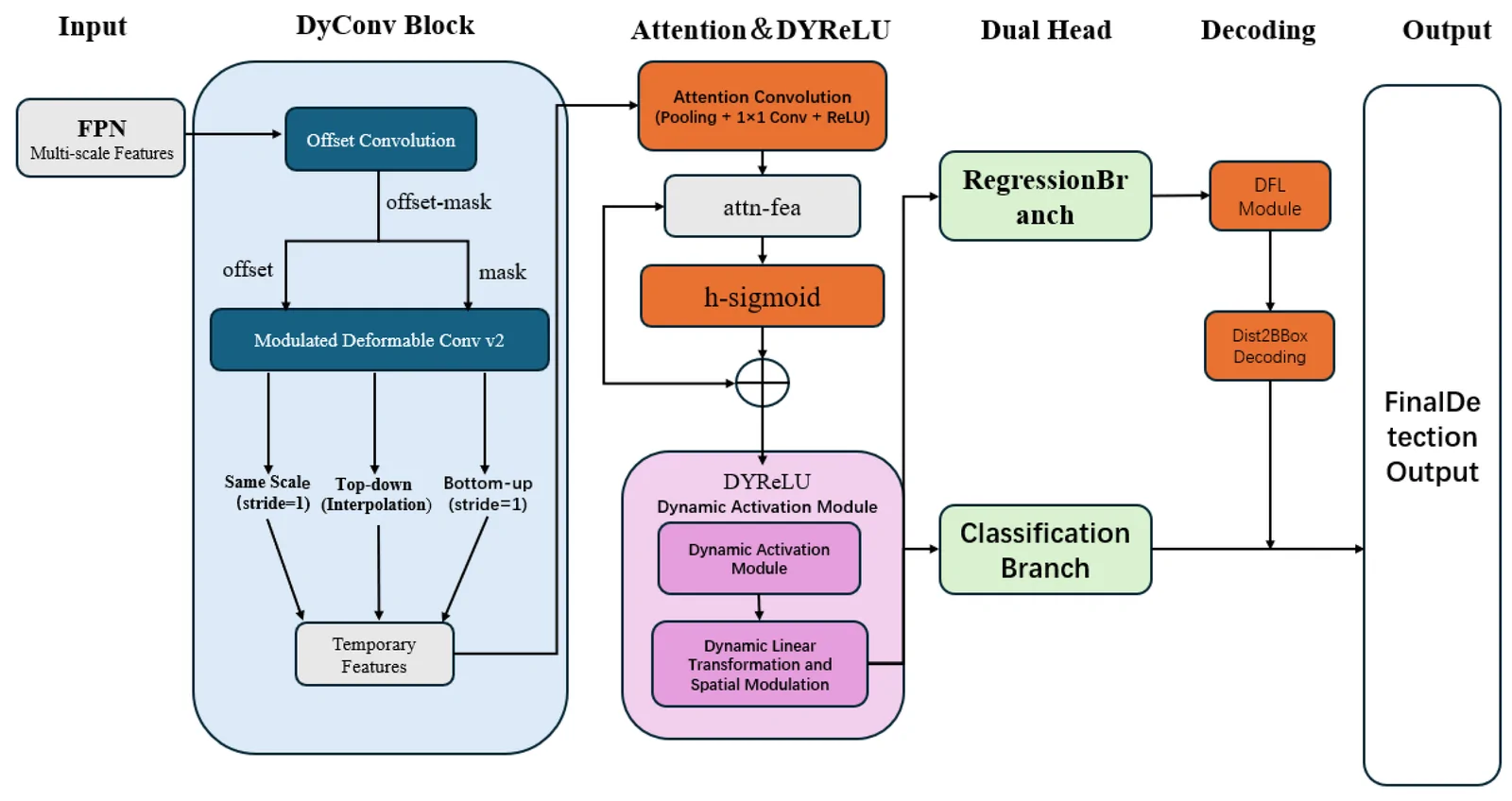
Architecture of the DyHead detection network. The framework integrates feature pyramid inputs with a dynamic convolution-based feature enhancement module, followed by attention-based adaptive feature refinement and DYReLU activation. The dynamic detection head adaptively adjusts feature responses across-scale, spatial, and task dimensions, thereby improving the detection of small, dense, and partially occluded pepper pests.

To improve the adaptability of feature representation before prediction, DyHead (Dynamic Head) is incorporated into the detection head of HCFD-YOLOv8 (see Figure 6). Unlike static detection heads that use fixed feature responses, DyHead dynamically adjusts feature representations by considering different object scales, spatial locations, and semantic information. This design enables more flexible feature refinement and enhances the perception capability of the detector under complex agricultural environments.

The main advantage of DyHead is that it employs dynamically learnable weights to adaptively fuse convolution responses from different receptive fields. This enables the detection head to automatically adjust its feature extraction process according to the semantic complexity of the input features. As a result, more discriminative and robust feature representations can be obtained for object detection tasks. As shown in Equation (3).

(3) $$F_{l}=\sum_{k\in N(l)}\alpha_{k}^{l}\cdot DConv_{k}(X^{k})$$

where *F*_l_ denotes the fused feature map at the *l*-th layer of DyHead, and (DConvk) represents the deformable convolution operation. α*_k_* is the weight coefficient of the *k*-th feature map when it is fused into the output feature of the *l*-th layer. It is adaptively learned by the dynamic weighting mechanism of DyHead according to the input features. *l* denotes the current target level, which usually refers to a feature map in the Feature Pyramid Network (FPN), and *X^k^* represents the input feature map at the *k*-th layer.

The dynamic weighting mechanism effectively improves the robustness of the network to target scale variations, shape deformation, and dense background interference. Compared with conventional static convolutions, it provides greater feature representation flexibility. In addition, DyHead introduces a cross-level context enhancement module to jointly model local details and global semantic information in both the spatial and channel domains. This enables the detection head to establish stable semantic relationships among features at different levels and improves the discriminability of small and blurred targets, as shown in Equation (4).

(4) $$Y=\max(a_{1}(X)\cdot X+b_{1}(X),a_{2}(X)\cdot X+b_{2}(X))$$

where *X* denotes the input feature map, and *Y* denotes the output feature map. *a*_1_(*X*) and *a*_2_(*X*) are input-dependent scaling coefficients, while *b*_1_(*X*) and *b*_2_(*X*) are input-dependent bias terms.

From the perspective of localization accuracy, DyHead improves detection performance by enhancing adaptive feature representation before bounding-box prediction. In complex pepper pest images, targets often exhibit large variations in scale and are frequently affected by partial occlusion from leaves and stems. Conventional static detection heads apply fixed feature transformations to different targets, which may limit effective feature alignment between predicted bounding boxes and ground-truth pest regions.

In contrast, DyHead dynamically refines feature responses across different feature levels, spatial locations, and task-specific branches. This adaptive mechanism enhances informative features associated with pest regions, strengthens spatial responses around object boundaries, and suppresses irrelevant background interference. Consequently, the regression branch receives more discriminative and spatially aligned representations, improving localization accuracy for small and densely distributed pests.

Within the proposed HCFD-YOLOv8 framework, DyHead serves as an adaptive prediction refinement component rather than an independent detection module. Its dynamic feature adjustment capability enables the detector to better handle variations in pest scale, shape, and occlusion conditions, making it suitable for pepper pest detection under complex agricultural environments.

#### 2.3.4. Inner-MPDIoU Loss Function

Traditional IoU-based regression losses, including GIoU [27], DIoU [28,29], and CIoU [30], improve bounding-box optimization by introducing additional geometric constraints based on overlap, enclosure, or distance information. However, when the predicted box and the ground-truth box have limited overlap or exhibit significant scale differences, these losses may provide insufficient geometric guidance during the early regression stage. This limitation can affect localization stability, particularly for small objects with irregular boundaries and dense distributions.

To enhance geometric consistency in the regression process, Inner-MPDIoU (Inner Multi-Perspective Distance IoU) is incorporated into the regression branch of HCFD-YOLOv8. Unlike conventional IoU-based losses that mainly rely on global box-level constraints, Inner-MPDIoU introduces additional geometric relationships to provide more comprehensive localization guidance. This design aims to improve bounding-box regression accuracy and convergence stability for pepper pests under complex agricultural conditions.

Let $B_{p}$ and $B_{g}$ denote the predicted bounding box and the ground-truth bounding box, respectively. Their center coordinates and scale parameters are defined as follows:

(5) $$B_{p}=(x_{p},y_{p},w_{p},h_{p}),\;\;\;B_{g}=(x_{g},y_{g},w_{g},h_{g})$$

The predicted bounding box $B_{p}$ is represented as

(6) $$x_{p}=\frac{x_{1}^{p}+x_{2}^{p}}{2}\;\;\;y_{p}=\frac{y_{1}^{p}+y_{2}^{p}}{2}$$

(7) $$w_{p}=x_{2}^{p}-x_{1}^{p}\;\;\;h_{p}=y_{2}^{p}-y_{1}^{p}$$

The parameters of the ground-truth bounding box are defined similarly and are omitted for brevity.

According to the above definitions, the IoU is calculated as follows:

(8) $$IoU(B_{p},B_{g})=\frac{\left|B_{p}\cap B_{g}\right|}{\left|B_{p}\cup B_{g}\right|}$$

To further enhance geometric consistency, regression constraints are constructed within the overlapping region of the predicted box and the ground-truth box. This region is referred to as the Inner Box and is defined as follows:

(9) $$B_{inner}=B_{p}\cap B_{g}$$

Furthermore, the Inner-IoU is calculated based on the Inner Box and can be expressed as follows:

(10) $$IoU_{inner}=\frac{\left|B_{inner}\right|}{\left|B_{p}\cup B_{g}\right|}$$

**Figure 7:**
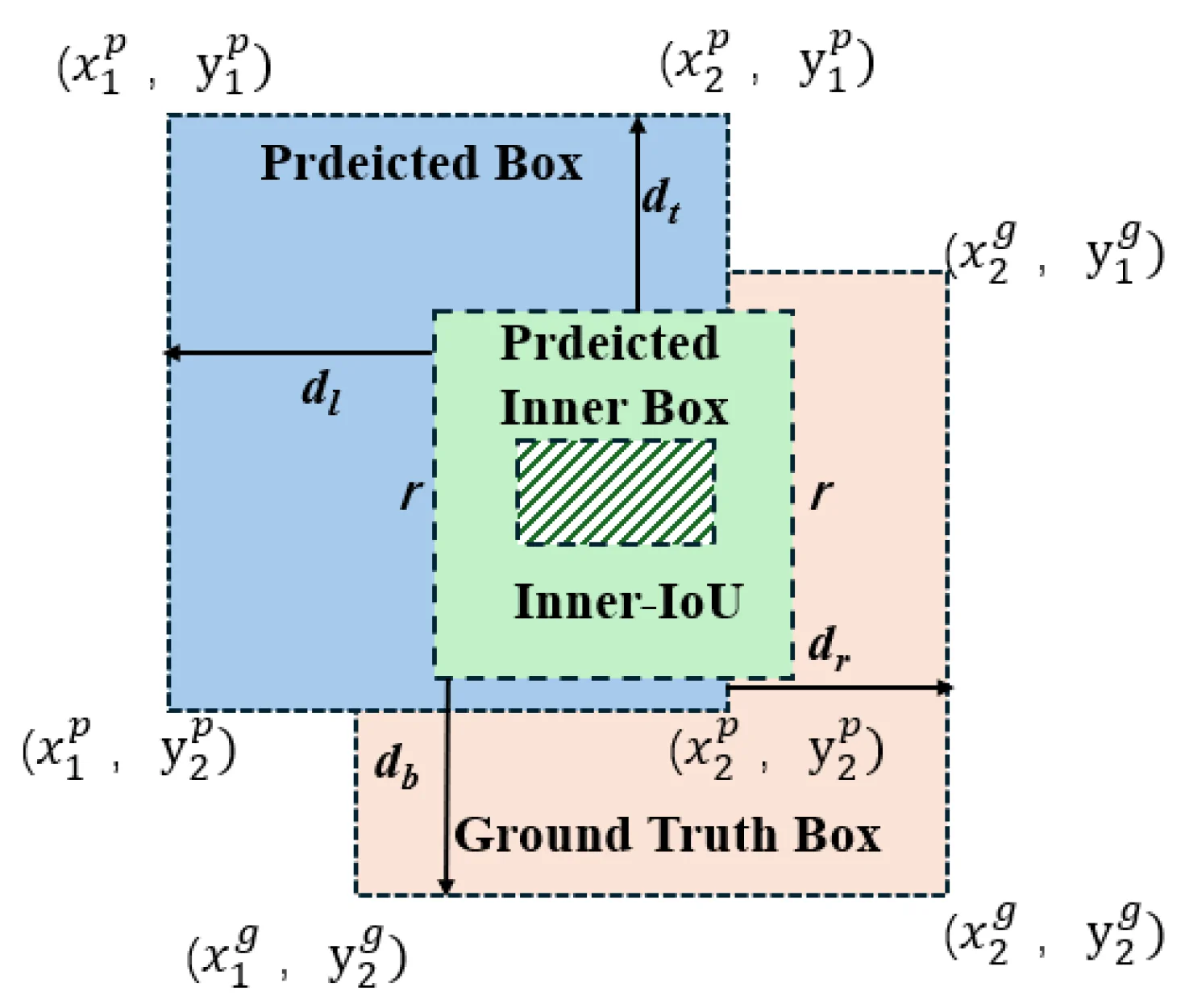
Schematic illustration of the Inner-MPDIoU loss function. The figure depicts the geometric relationship between the predicted bounding box, ground-truth box, and inner bounding box, where the loss is computed based on the intersection-over-union within the inner region to improve localization accuracy and training stability.

As illustrated by the green dashed box in Figure 7, $B_{inner}$ represents the internal reference region used for geometric constraint calculation between the predicted bounding box and the ground-truth bounding box. The green shaded area indicates the internal geometric region involved in regression optimization. Compared with conventional MPDIoU [31], which mainly relies on global box-level geometric relationships, Inner-MPDIoU introduces internal geometric constraints to provide additional localization guidance. This strategy can maintain more informative gradient feedback when the overlap between predicted and ground-truth boxes is limited, which is beneficial for small-object detection scenarios with inaccurate initial localization.

To further characterize the boundary alignment between predicted and ground-truth boxes from multiple directions, four internal boundary distances are defined along the left, right, top, and bottom directions. As illustrated by the arrows $d_{l}$, $d_{r}$, $d_{t}$, and $d_{b}$ in Figure 7, these multi-perspective internal boundary distances are formulated as follows:

(11) $$d_{b}^{inner}=\frac{1}{4}\sum_{k\in (l,r,t,b)}\frac{\left|\delta_{k}^{p}-\delta_{k}^{g}\right|}{\max(\delta_{k}^{g},\varepsilon )}$$

Here, $\delta_{k}^{p}$ and $\delta_{k}^{g}$ denote the distances from the center to the boundary along direction *k* for the predicted box and the ground-truth box, respectively, and ε is a tiny constant introduced to prevent numerical instability. As shown in the figure, the arrows in the left and right directions correspond to $\delta_{l}$ and $\delta_{r}$, and those in the top and bottom directions correspond similarly.

To avoid the uneven influence of center offset penalties for objects of different scales, the center distances are normalized based on the scale of the internal region. The internal center distance is defined as:

(12) $$d_{c}^{inner}=\frac{{\left\|\left(x_{p},y_{p})-(x_{g},y_{g}\right)\right\|}^{2}}{{\left(w_{inner}{)}^{2}+(h_{inner}\right)}^{2}}$$

As indicated by scale *r* in Figure 7, this scale reflects the geometric extent of the valid internal region. It enables the center offset penalty to focus more on the truly regressable area rather than the outer enclosing box.

By combining the IoU term, the internal center offset constraint, the multi-view internal boundary distances, and the shape consistency term, the final Inner-MPDIoU bounding box regression loss is defined as follows:

(13) $$L_{Inner-MPDIOU}=1-IoU+\lambda_{c}d_{c}^{inner}+\lambda_{b}d_{b}^{inner}+\lambda_{s}d_{s}^{inner}$$

Inner-MPDIoU establishes a multi-perspective distance metric based on internal geometric relationships between predicted and ground-truth bounding boxes. It explicitly considers geometric information within the internal region, including center displacement, boundary alignment, and shape consistency. Unlike conventional distance-based regression metrics that mainly rely on global box-level constraints, the internal geometric modeling strategy of Inner-MPDIoU provides additional localization guidance when the overlap between predicted and ground-truth boxes is limited.

By introducing internal geometric constraints, Inner-MPDIoU alleviates insufficient gradient information under low-overlap conditions, especially during the early stage of regression optimization. Furthermore, the multi-perspective geometric representation provides more detailed shape alignment information, enabling more effective optimization of bounding-box boundaries and improving localization robustness.

Compared with MPDIoU, Inner-MPDIoU further enhances the modeling of internal geometric consistency during regression. This design facilitates smoother localization optimization and improves the adaptability of the detector to challenging targets, including small objects, densely distributed pests, and objects with large aspect-ratio variations. Therefore, incorporating Inner-MPDIoU into HCFD-YOLOv8 provides stronger localization constraints and contributes to more accurate bounding-box prediction.

Inner-MPDIoU enhances localization optimization by introducing additional internal geometric constraints during bounding-box regression. Conventional IoU-based losses mainly rely on the overlap relationship between predicted and ground-truth boxes. However, when the overlap is limited, especially for small pest targets with partial occlusion, the available geometric guidance may become insufficient during regression optimization.

To address this limitation, Inner-MPDIoU incorporates internal geometric constraints between the predicted and ground-truth bounding boxes. Specifically, the internal center-distance constraint improves the alignment of box centers, while the multi-view boundary-distance constraints guide the predicted box toward the ground-truth box from the left, right, top, and bottom directions. In addition, the shape-consistency constraint reduces geometric discrepancies in width and height proportions.

These complementary constraints provide more detailed localization supervision compared with conventional IoU-based regression losses. As a result, Inner-MPDIoU offers more effective geometric guidance for challenging targets, including small, densely distributed, and overlapping pepper pests, thereby improving boundary alignment and localization robustness.

#### 2.3.5. HCFD-YOLO Model

**Figure 8:**
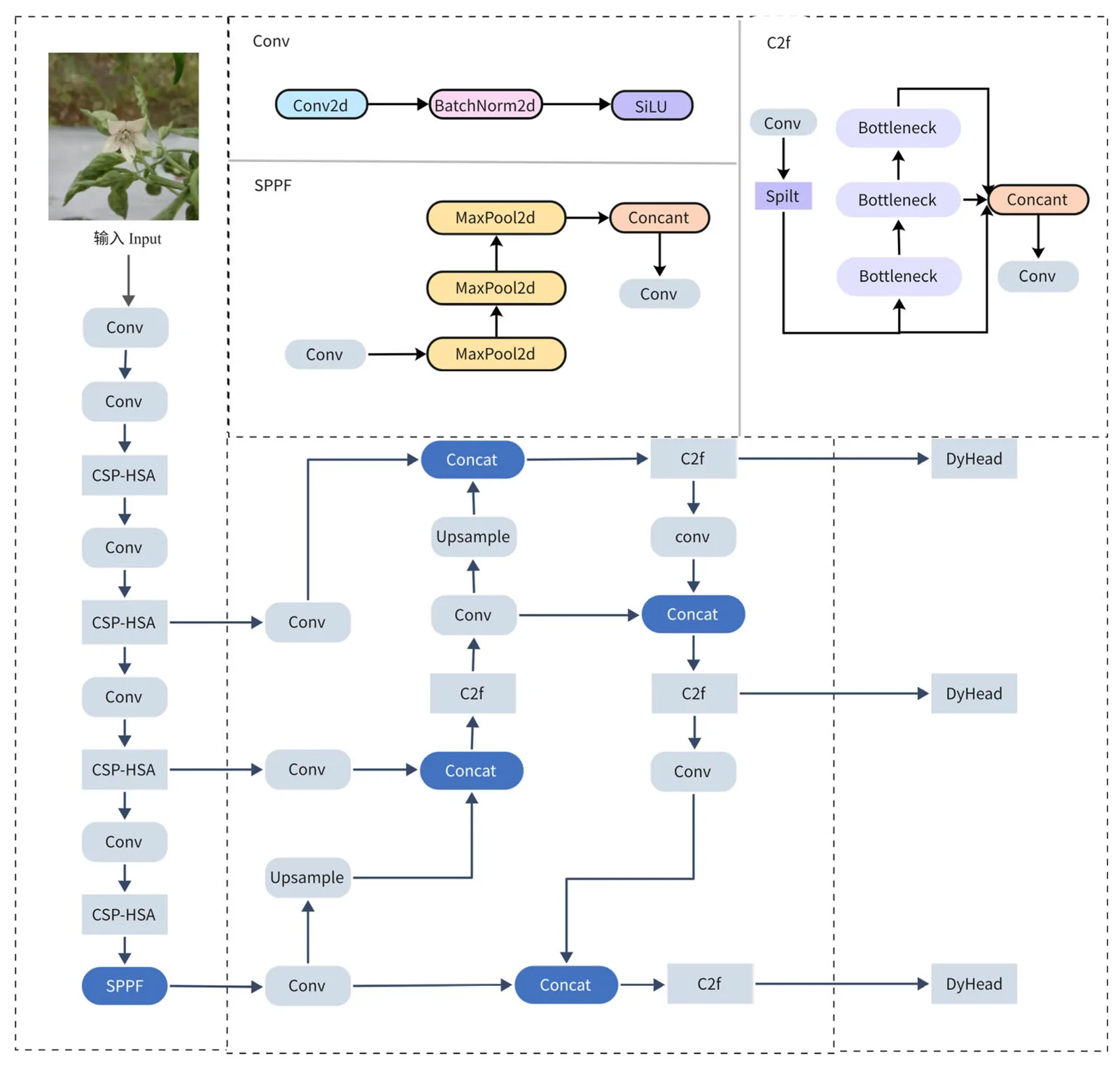
Overall architecture of HCFD-YOLOv8, showing the CSP-HSA backbone, CCFM neck, and DyHead detection head. Inner-MPDIoU is used for bounding-box regression during training.

This study develops HCFD-YOLOv8, a lightweight small-object pepper pest detection framework based on YOLOv8n, to address the challenges of small target size, large variations in appearance and scale, and complex background interference in practical pepper cultivation environments. The overall architecture of the proposed framework is illustrated in Figure 8.

First, CSP-HSA replaces the original C2f module in the backbone. By integrating cross-stage feature reuse and lightweight spatial-channel feature refinement, it enhances discriminative feature extraction while reducing redundant computation. This design improves the representation capability of small and densely distributed pepper pests under complex agricultural backgrounds.

Second, CCFM is introduced into the neck network to strengthen cross-scale feature interaction. It facilitates information exchange between high-resolution spatial details and high-level semantics, improving multi-scale feature consistency for pepper pests with different sizes and appearances.

Third, a Dynamic Head (DyHead) mechanism is incorporated into the detection head. By adaptively refining feature responses according to object scales, spatial locations, and semantic information, DyHead improves the flexibility of feature representation before prediction and enhances the detection performance for targets with scale variation and occlusion.

Finally, the original CIoU loss is replaced with Inner-MPDIoU for bounding-box regression. By introducing internal geometric constraints, Inner-MPDIoU provides more effective localization guidance and improves regression stability for small, dense, and overlapping pepper pest targets.

### 2.4. Experimental Platform and Configuration

All experiments were conducted on a cloud-computing platform. The software environment consisted of Python 3.9.7, PyTorch 1.12.1, and CUDA 11.6 for GPU acceleration. The hardware environment included a 12-core virtual CPU instance based on an Intel(R) Xeon(R) Platinum 8352 V processor, a virtual GPU instance with 32 GB of memory, and 90 GB of system memory (Intel Corporation, Santa Clara, CA, USA). Hardware and software configurations of the experimental platform are shown in Table 4.

All comparative models were trained and evaluated under identical hardware and software configurations. To ensure a fair comparison, all input images were uniformly resized to 640 × 640 pixels during training and inference. Since the cloud-computing platform provided only the allocated virtual GPU specification without revealing the exact underlying physical GPU model, the accelerator configuration is reported as a 32 GB virtual GPU instance.

### 2.5. Model Evaluation Metrics

To comprehensively evaluate the detection accuracy and computational efficiency of the proposed HCFD-YOLOv8 framework for pepper pest detection, several performance and efficiency metrics were adopted. Precision (P), Recall (R), mAP50, and mAP50:95 were used to evaluate detection performance, while the number of parameters, floating-point operations (FLOPs), model weight-file size, and inference throughput were used to assess model complexity and computational efficiency.

Precision represents the proportion of correctly detected positive instances among all predicted positive instances, whereas Recall indicates the proportion of actual positive instances correctly identified by the model. The F_1_-score is the harmonic mean of Precision and Recall, providing a balanced measurement of detection performance. The mAP50 metric represents the mean average precision calculated at an IoU threshold of 0.5. The mAP50:95 metric is obtained by averaging AP values over IoU thresholds from 0.5 to 0.95 with an interval of 0.05, following the evaluation protocol.

In addition to parameter number and FLOPs, model weight-file size and inference throughput were considered supplementary indicators of engineering efficiency. The model weight-file size refers to the disk storage occupied by the saved trained model weights and should not be interpreted as runtime memory consumption. Inference throughput was measured in frames per second (FPS), representing the number of images processed per second under the evaluated cloud-server environment.

To ensure a fair comparison, all models were evaluated using the same hardware and software environment, input resolution, and inference settings. The reported FPS values represent the processing throughput achieved on the evaluated cloud-computing platform and should not be directly interpreted as the deployment performance on embedded agricultural devices. The corresponding evaluation formulas are defined as follows:

(14) $$P=\frac{\mathrm{TP}}{\mathrm{TP}+\mathrm{FP}}$$

(15) $$R=\frac{\mathrm{TP}}{\mathrm{TP}+\mathrm{FN}}$$

where TP denotes the number of correctly detected pest instances, FP denotes the number of incorrectly predicted instances, including false detections from background regions or incorrect categories, and FN denotes the number of pest instances that are not detected by the model.

When the class distribution is imbalanced, mAP provides a more comprehensive evaluation of detection performance by considering both classification confidence and localization accuracy. As shown in Equation (16), mAP is calculated by integrating the area under the precision–recall (PR) curve for each category and then averaging the results over all categories. Specifically, the mean value is obtained according to the number of categories k.

(16) $$\mathrm{mAP}=\frac{1}{k}\sum_{i=1}^{k}\int_{0}^{1}P_{i}(R_{i})dR_{i}$$

Model weight-file size and FPS were used as supplementary engineering-efficiency indicators. Model weight-file size refers to the disk storage occupied by the saved best-performing PyTorch weight file. FPS is defined as the number of images processed per second and can be expressed as:

(17) $$\mathrm{FPS}=\frac{N}{T}$$

where N denotes the total number of processed images and T denotes the corresponding processing time in seconds.

## 3. Results

### 3.1. Ablation Experiment

Each configuration was independently trained three times under identical dataset splits and training settings, and the reported detection metrics are presented as mean ± standard deviation. Given the limited number of repeated runs (*n* = 3 for each configuration), formal statistical significance testing was not performed. Therefore, the reported mean ± standard deviation values characterize run-to-run variability, and the observed differences should be interpreted as descriptive performance gains under the current experimental conditions rather than as statistically confirmed superiority. To systematically isolate the individual and interactive effects of CSP-HSA, CCFM, DyHead, and Inner-MPDIoU, a complete $2^{4}$ factorial ablation study was conducted, covering all 16 possible module combinations. All configurations were trained and evaluated using identical dataset splits, training settings, and evaluation protocols. The experimental results are presented in Table 5.

As shown in Table 5, the four components introduced in HCFD-YOLOv8 contribute differently to detection performance and computational efficiency. The results demonstrate that each component addresses a specific limitation of the baseline YOLOv8n architecture rather than providing redundant improvements.

Among the individual modifications, DyHead yields the largest single improvement in detection performance. Compared with the YOLOv8n baseline, introducing DyHead increases Precision from 84.0% to 90.1%, Recall from 82.8% to 88.5%, mAP50 from 87.1% to 90.8%, and mAP50:95 from 54.3% to 54.9%. These improvements indicate that adaptive feature refinement before prediction enhances the perception of small and densely distributed pepper pests. However, this accuracy improvement requires additional computational resources, with parameters and FLOPs increasing from 3.00 M and 7.5 G to 3.90 M and 8.5 G, respectively.

CSP-HSA demonstrates a better trade-off between feature enhancement and computational efficiency. When independently incorporated into YOLOv8n, CSP-HSA improves Precision and Recall to 85.6% and 84.6%, respectively, while reducing parameters from 3.00 M to 2.54 M and FLOPs from 7.5 G to 7.2 G. Furthermore, the CSP-HSA–DyHead combination achieves the highest detection performance among the evaluated partial configurations, with Precision, Recall, mAP50, and mAP50:95 reaching 91.6%, 90.4%, 92.5%, and 56.3%, respectively. These results suggest that the lightweight feature extraction capability of CSP-HSA provides complementary fine-grained representations for DyHead-based adaptive prediction.

CCFM mainly contributes to efficient cross-scale feature interaction and model compactness. When applied independently, CCFM reduces parameters from 3.00 M to 2.89 M and FLOPs from 7.5 G to 6.8 G, while maintaining comparable Precision and Recall to the baseline. Although its individual contribution to detection accuracy is limited, combining CCFM with DyHead increases mAP50 from 90.8% to 91.2% while reducing parameters from 3.90 M to 3.12 M and FLOPs from 8.5 G to 7.6 G. The slight decrease in mAP50:95 indicates that CCFM primarily improves feature efficiency and cross-scale representation rather than directly optimizing localization accuracy.

Inner-MPDIoU modifies the regression objective without introducing additional parameters or inference-time computational cost. When added to the baseline model, Inner-MPDIoU increases mAP50 from 87.1% to 87.5% and mAP50:95 from 54.3% to 54.8%. In the CCFM-based configuration, Inner-MPDIoU further improves mAP50:95 from 53.1% to 54.8%. Moreover, integrating Inner-MPDIoU into the CSP-HSA–DyHead–CCFM configuration improves Recall from 84.5% to 89.8%, mAP50 from 90.5% to 92.0%, and mAP50:95 from 53.4% to 55.2%. These results indicate that the effectiveness of Inner-MPDIoU depends on the quality of feature representations provided by the backbone, neck, and detection head, rather than acting as an independent additive improvement.

The complete HCFD-YOLOv8 framework achieves Precision of 90.9%, Recall of 89.8%, mAP50 of 92.0%, and mAP50:95 of 55.2%, with only 2.43 M parameters and 6.5 G FLOPs. Compared with YOLOv8n, the proposed model improves Precision, Recall, mAP50, and mAP50:95 by 6.9, 7.0, 4.9, and 0.9 percentage points, respectively, while reducing parameters and FLOPs by 19.0% and 13.3%.

Compared with the CSP-HSA–DyHead configuration, the complete model reduces the parameter count and FLOPs by approximately 29.4% and 20.7%, respectively, while Precision, Recall, mAP50, and mAP50:95 decrease by 0.7, 0.6, 0.5, and 1.1 percentage points, respectively. Therefore, the complete model provides a favorable compromise between detection accuracy and computational efficiency for lightweight applications.

### 3.2. Comparative Experiments

**Figure 9:**
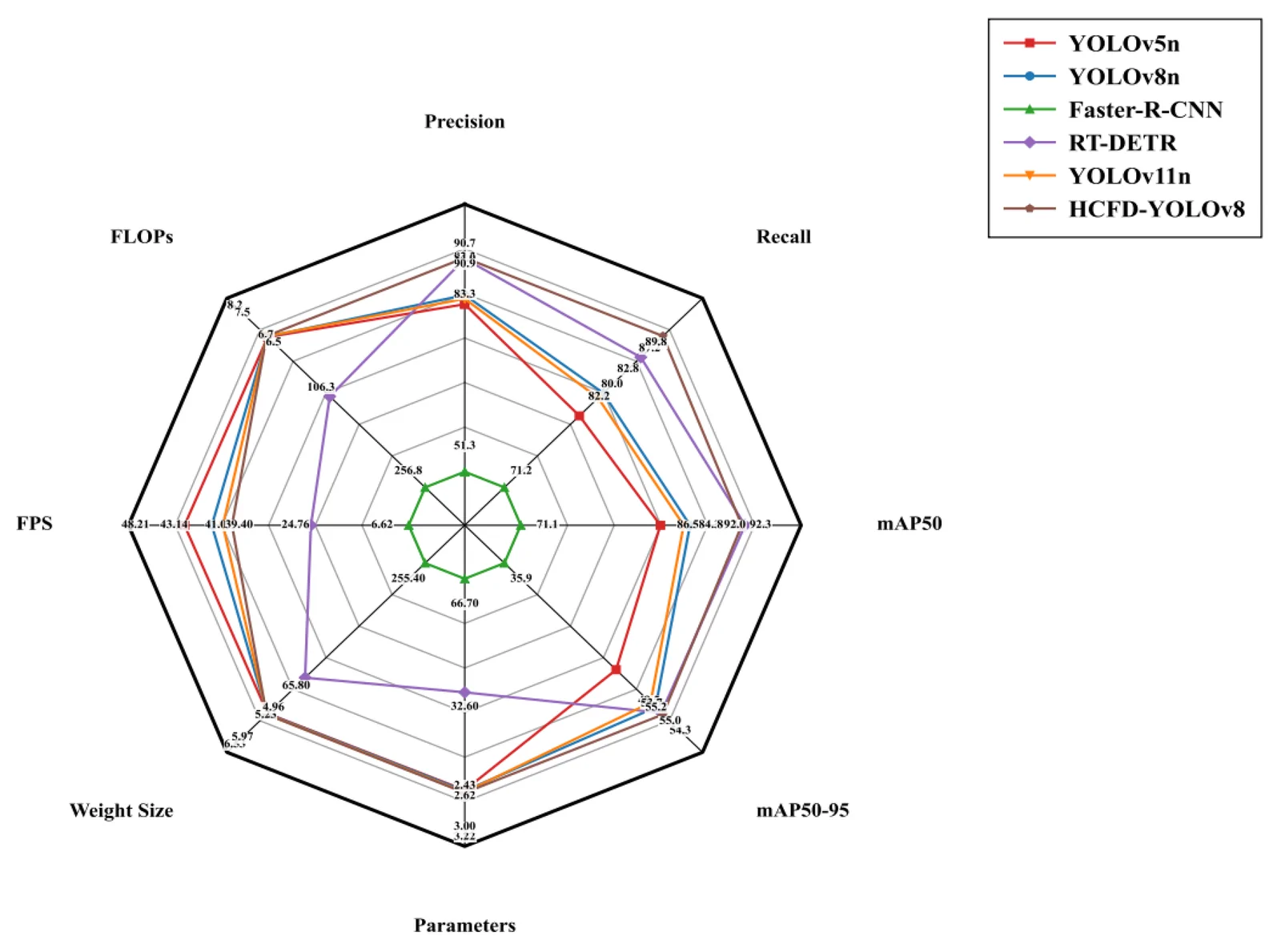
Radar chart comparison of different object detection models in terms of Precision, Recall, mAP50, mAP50:95, Parameters, Weight, FPD, and FLOPs. The proposed HCFD-YOLOv8 achieves the best overall balance between detection accuracy and computational efficiency compared with YOLOv5n, YOLOv8n, Faster R-CNN, RT-DETR, and YOLOv11n.

Under the same experimental conditions, the proposed improved YOLOv8n model was compared with several mainstream object detection algorithms, including Faster R-CNN, RT-DETR, and different models of the YOLO series. The comparison results are presented in Table 6 and Figure 9.

The comparative results demonstrate that HCFD-YOLOv8 achieves a favorable balance between detection accuracy and computational efficiency among the evaluated models. Compared with the YOLOv8n baseline, HCFD-YOLOv8 improves Precision from 84.0% to 90.9%, Recall from 82.8% to 89.8%, mAP0.5 from 87.1% to 92.0%, and mAP0.5:0.95 from 54.3% to 55.2%, corresponding to increases of 6.9, 7.0, 4.9, and 0.9 percentage points, respectively. Meanwhile, the proposed model reduces the number of parameters from 3.00 M to 2.43 M, the weight-file size from 5.97 MB to 4.96 MB, and FLOPs from 7.5 G to 6.5 G.

Compared with YOLOv11n, HCFD-YOLOv8 further improves Precision, Recall, mAP50, and mAP50:95 by 7.6, 7.6, 5.5, and 1.5 percentage points, respectively. At the same time, it reduces the parameter count from 2.62 M to 2.43 M, weight-file size from 5.23 MB to 4.96 MB, and FLOPs from 6.7 G to 6.5 G. Although the inference speed of HCFD-YOLOv8 (39.40 FPS) is lower than those of YOLOv5n, YOLOv8n, and YOLOv11n, it still maintains competitive processing throughput on the evaluated cloud-server platform while providing substantially improved detection accuracy.

RT-DETR achieves a slightly higher mAP50 value (92.3%) than HCFD-YOLOv8 (92.0%). However, HCFD-YOLOv8 provides higher Precision, Recall, and mAP50:95 with substantially lower computational requirements, using only 2.43 M parameters, a 4.96 MB weight file, and 6.5 G FLOPs. In comparison, RT-DETR requires considerably greater model complexity. Faster R-CNN also introduces substantially higher computational costs while achieving lower detection performance.

To further evaluate the lightweight characteristics of the proposed framework, YOLOv11n was included as an additional lightweight baseline for comparison. Its performance was analyzed together with the comparative experiments presented in Table 6. It can be seen that YOLOv11n achieved Precision, Recall, mAP50, and mAP50:95 values of 83.3%, 82.2%, 86.5%, and 53.7%, respectively, with 2.62 M parameters and 6.7 G FLOPs. In comparison, HCFD-YOLOv8 achieved substantially higher detection performance, with Precision, Recall, mAP50, and mAP50:95 values of 90.9%, 89.8%, 92.0%, and 55.2%, respectively, while requiring only 2.43 M parameters and 6.5 G FLOPs. These results demonstrate that HCFD-YOLOv8 improves detection capability over YOLOv11n while maintaining a comparable or even lower computational burden.

Therefore, compared with both YOLOv8-based lightweight backbone variants and the more recent YOLOv11n detector, the proposed framework achieves a favorable balance between detection accuracy and computational efficiency, indicating its potential for resource-constrained pepper pest monitoring applications. Overall, these results indicate that HCFD-YOLOv8 provides an effective balance among detection accuracy, model compactness, and computational efficiency. The proposed framework demonstrates strong potential for lightweight pepper pest monitoring applications, particularly in scenarios where computational resources are limited.

### 3.3. Comparison with Other Lightweight Modules

**Figure 10:**
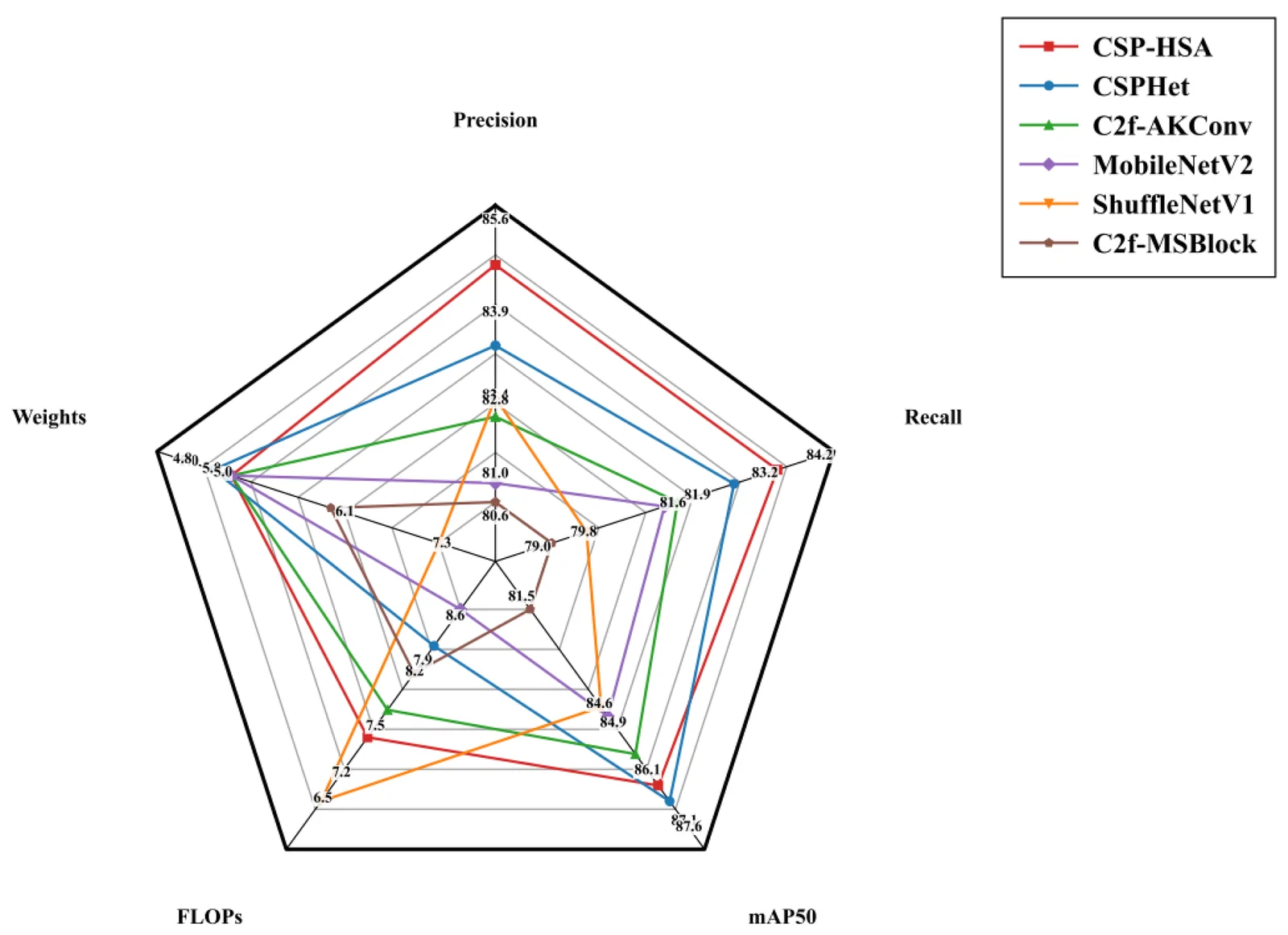
Radar chart comparison of different backbone networks in terms of Precision, Recall, mAP50, and computational efficiency metrics (FLOPs and model weights). The proposed CSP-HSA consistently achieves the best overall balance between detection accuracy and computational cost compared with CSPHet, C2f-AKConv, MobileNetV2, ShuffleNetV1, and C2f-MSBlock.

Under identical training configurations to the YOLOv8n baseline, different lightweight backbone designs were evaluated while keeping the remaining network structures and experimental settings unchanged. The training results were compared with those of the original YOLOv8n model to assess the feature extraction capability and computational efficiency of the proposed CSP-HSA module. To quantitatively compare the feature extraction capability and computational efficiency of different backbone architectures, six lightweight backbone designs, including CSP-HSA, CSPHet [32], C2f-AKConv [33], MobileNetV2 [34], ShuffleNetV1 [35], and C2f-MSBlock [36], were evaluated under identical experimental settings. The comparison results are presented in Table 7 and Figure 10.

Among the evaluated backbone structures, CSP-HSA achieved the most favorable balance between detection accuracy and computational efficiency. It obtained Precision, Recall, and mAP50 values of 85.6%, 84.2%, and 87.1%, respectively, while requiring only 7.2 G FLOPs and a 5.0 MB weight-file size. These results indicate that CSP-HSA can enhance feature representation without introducing substantial computational overhead.

In comparison, MobileNetV2 adopts a mature lightweight architecture; however, it required 8.6 G FLOPs while achieving a relatively lower mAP50 of 84.9%. This suggests that excessive lightweight compression may reduce feature representation capability under complex agricultural conditions. ShuffleNetV1 achieved lower computational cost (6.5 G FLOPs), but its Recall decreased to 79.8%, indicating a higher probability of missed detections. C2f-MSBlock also maintained a compact structure but showed limited detection performance, with an mAP50 of 81.5%, suggesting insufficient feature extraction capability for complex pest detection scenarios.

Compared with CSP-HSA, CSPHet achieved a slightly higher mAP50 value (87.6% vs. 87.1%). However, its Precision and Recall were lower, reaching 83.9% and 83.2%, respectively. Moreover, CSPHet required higher computational cost, with 8.2 G FLOPs compared with 7.2 G FLOPs for CSP-HSA, although it achieved a slightly smaller weight-file size of 4.8 MB. These results indicate that CSPHet obtains marginal improvement in mAP50 at the expense of increased computational complexity and reduced detection stability, highlighting the importance of balancing accuracy and efficiency in lightweight pest detection models.

Overall, the CSP-HSA backbone provides an effective trade-off among detection accuracy, model compactness, and computational cost. Its enhanced feature extraction capability enables more discriminative representations under complex background conditions, providing stronger feature support for subsequent detection stages. These results demonstrate the effectiveness of CSP-HSA as the backbone component of the proposed HCFD-YOLOv8 framework.

### 3.4. Model Visualization Analysis

**Figure 11:**
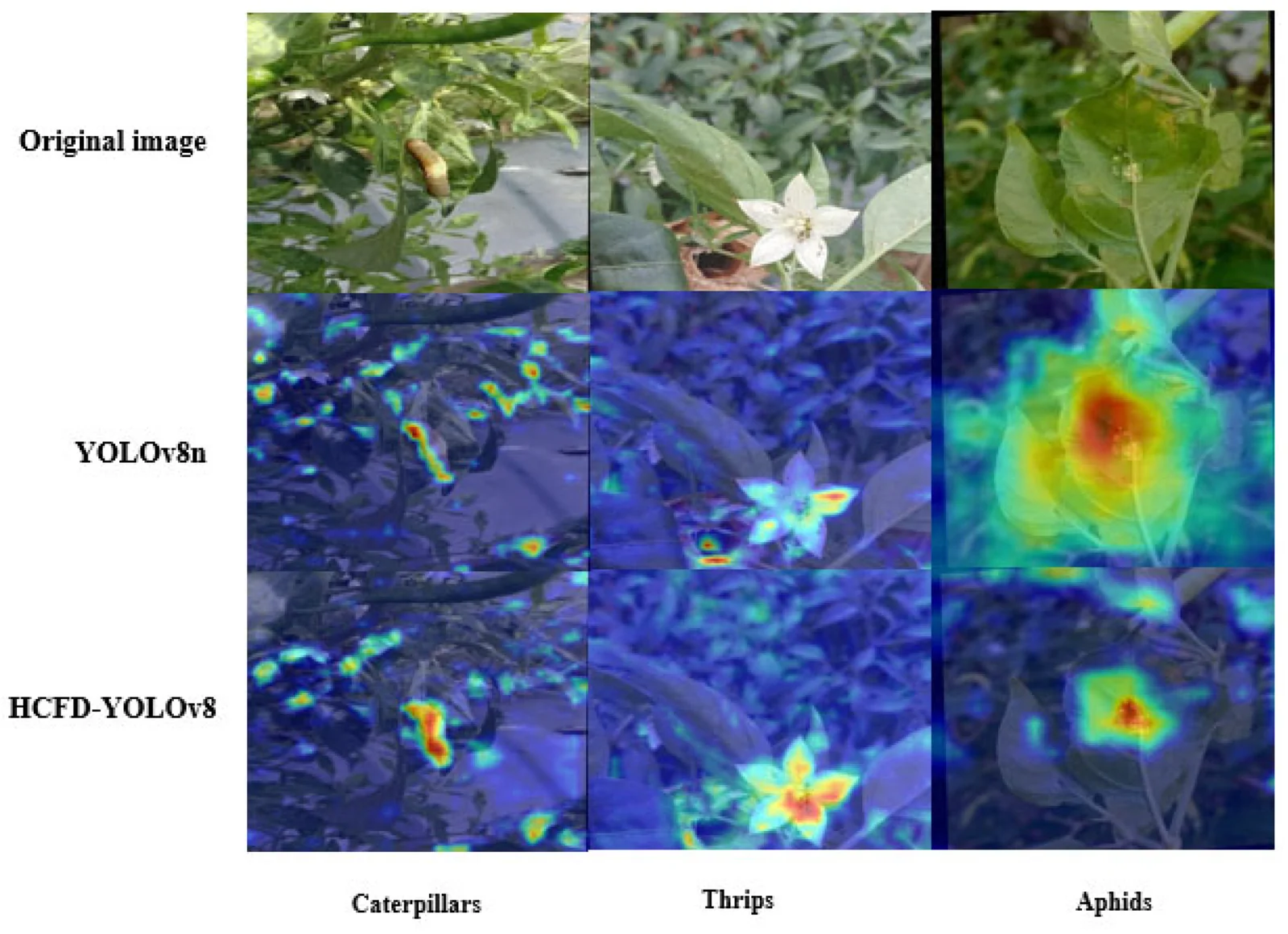
Comparison of activation heatmaps generated by YOLOv8n and HCFD-YOLOv8 for caterpillars, thrips, and aphids. In the selected examples, HCFD-YOLOv8 exhibits more spatially concentrated activation around visible pest regions.

A heatmap is a visualization approach that represents the intensity of model feature responses through color variations. Generally, brighter regions indicate stronger activation responses, reflecting areas that contribute more to the feature extraction and prediction process. Figure 11 presents the heatmap visualizations of HCFD-YOLOv8 and the baseline YOLOv8n model for pepper pest detection.

Figure 11 presents the feature activation maps generated by HCFD-YOLOv8 and the baseline YOLOv8n model for three representative pepper pest categories: caterpillars, thrips, and aphids. Compared with YOLOv8n, HCFD-YOLOv8 exhibits more spatially concentrated activation responses around visible pest regions. These qualitative observations suggest improved spatial focus of the complete framework in the selected examples. However, because HCFD-YOLOv8 integrates CSP-HSA, CCFM, DyHead, and Inner-MPDIoU, the visualization reflects their combined effects and cannot isolate the contribution of any individual component, including the HSA unit.

**Figure 12:**
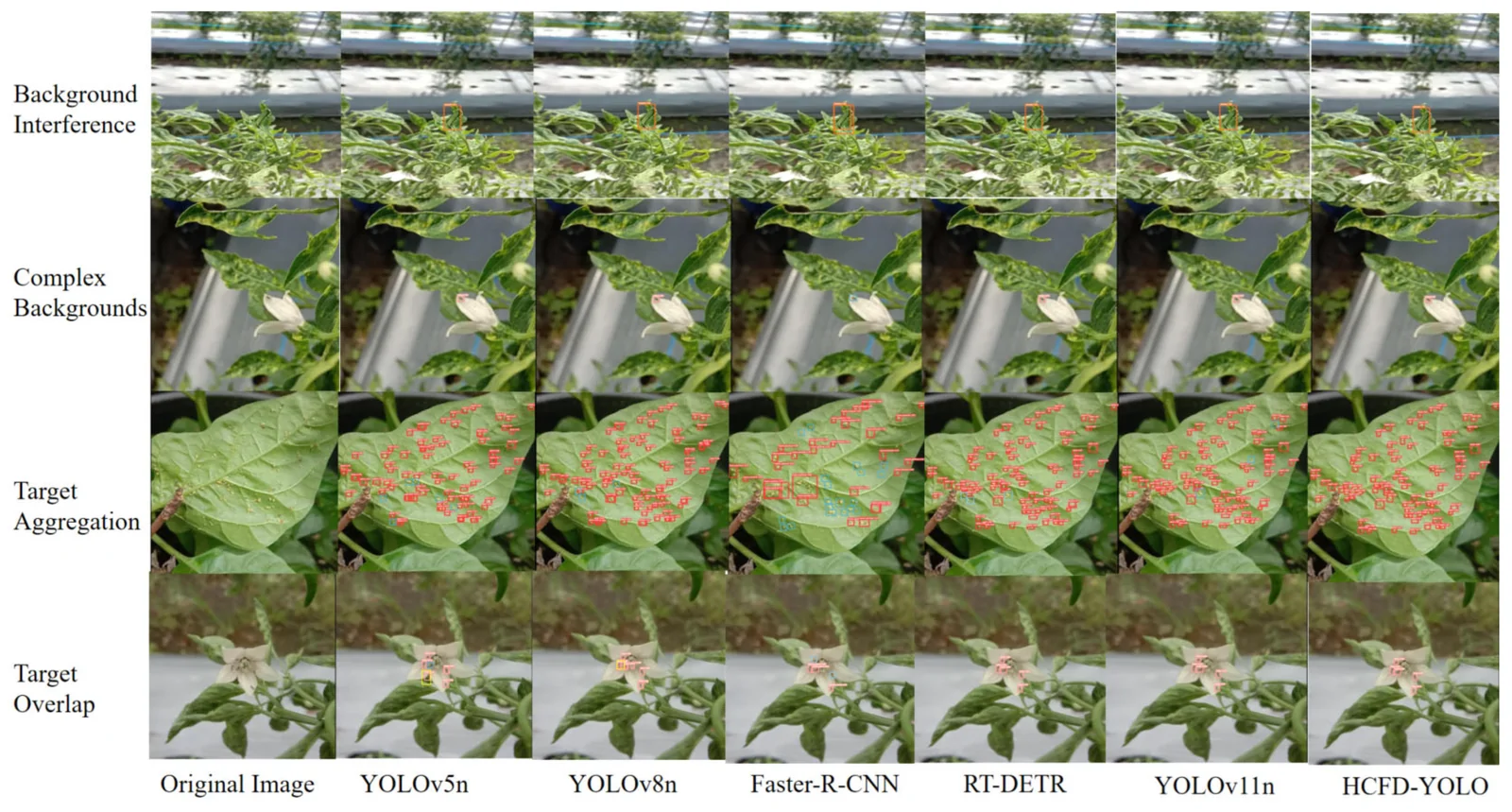
Qualitative comparison of detection results produced by different detectors under four challenging scenarios: background interference, complex backgrounds, target aggregation, and target overlap.

To further investigate the detection capability of HCFD-YOLOv8 under small-target and complex-scene conditions, additional qualitative experiments were conducted. The corresponding detection results are presented in Figure 12.

As shown in Figure 12, YOLOv8n exhibits several missed detections in the selected examples involving complex backgrounds, dense aggregation, and target overlap. In contrast, HCFD-YOLOv8 detects more pest instances and produces more complete detections in these examples. These qualitative observations suggest that the proposed framework can better emphasize pest-related features under challenging conditions.

**Figure 13:**
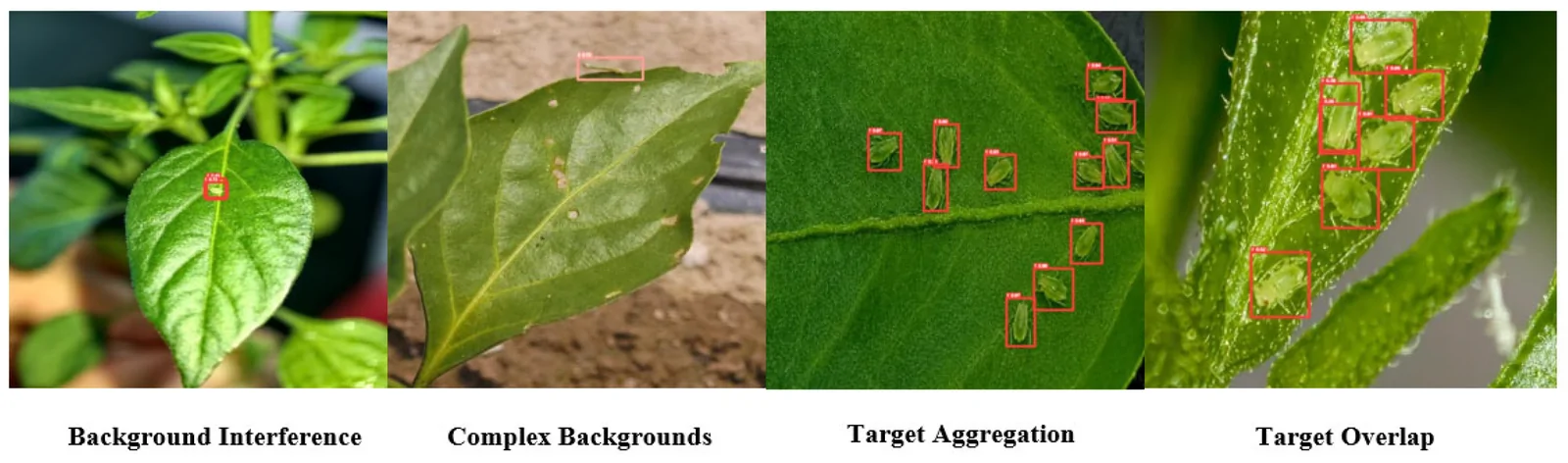
Detection results in real-world pepper cultivation scenes under different challenging conditions, including background interference, complex backgrounds, target aggregation, and target overlap. The visualization illustrates the detection performance of the proposed model under the representative agricultural conditions included in the present dataset.

It should be noted that the visualization results provide qualitative evidence of improved detection behavior rather than a comprehensive quantitative evaluation of model robustness. To further investigate the applicability of the proposed framework under practical cultivation conditions, additional experiments were conducted using images collected from real-world pepper cultivation environments. The corresponding detection results are presented in Figure 13.

As shown in Figure 13, HCFD-YOLOv8 can detect pest targets in representative open-field and greenhouse cultivation scenes involving background interference, complex vegetation environments, target aggregation, and target overlap. These results provide qualitative evidence that the proposed framework can maintain effective detection performance under the practical cultivation conditions represented by the collected images.

Nevertheless, the current evaluation does not independently consider more extreme environmental disturbances, such as adverse weather conditions, severe motion blur, rainfall, fog, or extreme illumination variations. Further validation under these challenging scenarios will be necessary to comprehensively assess the generalization capability of the model.

## 4. Discussion

The performance improvement of HCFD-YOLOv8 is attributed to the collaborative optimization of the proposed components at different stages of the detection pipeline. Pepper pest detection remains challenging because targets are typically small, densely distributed, partially occluded, and surrounded by complex agricultural backgrounds. These characteristics increase the difficulty of extracting discriminative features and achieving accurate localization, particularly for lightweight detection frameworks.

CSP-HSA plays a key role in enhancing feature representation while maintaining computational efficiency. By integrating lightweight convolution operations with hybrid spatial–channel attention, CSP-HSA improves feature interaction and supports the extraction of discriminative pest characteristics while maintaining computational efficiency. Meanwhile, CCFM strengthens cross-scale information exchange between spatial details and semantic representations, which is beneficial for detecting pests with different sizes and appearance variations. DyHead further improves adaptive feature refinement before prediction, while Inner-MPDIoU enhances localization optimization through additional geometric constraints during regression. Therefore, the improvement achieved by HCFD-YOLOv8 does not originate from a single module, but from the complementary cooperation among feature extraction, feature fusion, adaptive prediction, and localization optimization.

Recent studies have highlighted the importance of efficient feature representation and multi-scale information interaction in agricultural pest detection. ABF-YOLO11 [37] improved stored-grain pest detection by incorporating ADown, BiFPN, and an enhanced localization loss, with an emphasis on preserving fine-grained information and improving feature fusion. Similarly, OS-YOLO [38] integrated BiFPN, a Tiny Object Detection Layer (TODL), and dynamic convolution mechanisms to enhance small-object perception under complex storage environments. These studies demonstrate that effective feature extraction and multi-scale representation are essential factors for improving pest detection performance.

Different from these existing methods, HCFD-YOLOv8 was specifically designed for pepper pest detection under practical cultivation environments. The proposed framework simultaneously considers the characteristics of pepper pests, including small target size, dense distribution, background interference, and target overlap. Through the coordinated integration of CSP-HSA, CCFM, DyHead, and Inner-MPDIoU, the model improves feature discrimination, cross-scale interaction, adaptive prediction capability, and localization accuracy within a unified lightweight framework. This design enables HCFD-YOLOv8 to achieve improved detection performance while maintaining relatively low computational complexity, providing potential support for resource-efficient agricultural monitoring applications.

The visualization analysis further supports the effectiveness of the proposed framework. The heatmap results show that HCFD-YOLOv8 generates more spatially concentrated responses around pest-related regions in the selected examples. Nevertheless, these heatmaps reflect the behavior of the complete framework and do not independently validate a background-suppression effect of the HSA unit. In addition, qualitative detection results demonstrate improved detection behavior under challenging scenarios, including small targets, dense distributions, background interference, and target overlap. These observations indicate that the proposed optimization strategy enhances both feature representation and localization capability for pepper pest detection.

The improvement in localization accuracy is attributed to the complementary effects of DyHead and Inner-MPDIoU. DyHead provides spatially aligned and task-adaptive features, while Inner-MPDIoU introduces internal geometric constraints during bounding-box regression. Hence, they enhance feature alignment and localization optimization for small and overlapping pepper pests under complex backgrounds.

Although HCFD-YOLOv8 achieves promising detection performance, several limitations remain. First, the current dataset contains only three pest categories and was collected from relatively limited field and greenhouse environments. Although images with different plant appearances and environmental backgrounds were included to improve visual diversity, the dataset does not represent a complete year-round monitoring scenario. Data augmentation can improve robustness to certain simulated variations; however, it cannot fully replace large-scale data collection under diverse agricultural conditions. Therefore, systematic variations associated with seasons, weather conditions, and crop growth stages were not completely covered in the present study. In addition, all experiments were conducted using a single constructed pepper pest dataset, and the performance of HCFD-YOLOv8 on independent external datasets has not yet been validated. Consequently, the current findings should be interpreted within the scope of the dataset and experimental conditions evaluated in this study. Future work will focus on expanding the dataset with more pest categories, broader cultivation environments, and longer monitoring periods, as well as conducting external validation using independently collected datasets from different geographic regions, cultivation environments, seasons, imaging devices, and pest populations to more rigorously evaluate model generalizability.

Second, the engineering-efficiency evaluation was mainly based on parameter count, FLOPs, weight-file size, and FPS measured on a cloud-server platform. The reported weight-file size reflects storage requirements rather than runtime memory consumption, and embedded-device latency, energy consumption, and memory usage were not directly evaluated. Therefore, the current results demonstrate model compactness and deployment potential rather than completed edge-device validation. Future studies will investigate the performance of HCFD-YOLOv8 on representative agricultural edge platforms.

Third, although the dataset includes challenging conditions such as illumination variation, complex vegetation backgrounds, target-scale variation, dense aggregation, partial occlusion, and target overlap, it was not specifically designed for systematic stress testing under extreme conditions, including adverse weather, severe motion blur, rainfall, fog, and extreme illumination. Therefore, the current visualization results provide qualitative evidence under the evaluated conditions rather than comprehensive robustness validation across all agricultural environments.

Fourth, although each model configuration was independently trained three times and the corresponding metrics were reported as mean ± standard deviation, formal statistical significance testing was not conducted because of the limited number of repeated runs. Consequently, the observed performance gains represent descriptive numerical improvements under the current dataset split and experimental protocol, but they do not establish statistically significant superiority or guarantee that improvements of the same magnitude will be reproduced across other datasets and deployment conditions. Future studies with additional repeated runs and formal paired statistical comparisons are required to quantify the statistical uncertainty and robustness of these improvements more rigorously.

Overall, comparative experiments demonstrate that the performance improvement of HCFD-YOLOv8 does not result from simply replacing individual network components, but from coordinated optimization across feature extraction, feature fusion, adaptive prediction, and localization regression. CSP-HSA, CCFM, DyHead, and Inner-MPDIoU address different limitations within the detection pipeline and provide complementary improvements. Compared with YOLOv8n, YOLOv11n, and other lightweight backbone variants, HCFD-YOLOv8 achieves a favorable balance between detection accuracy and computational efficiency, indicating its potential for resource-efficient pepper pest monitoring applications.

## 5. Conclusions

In this study, a task-oriented lightweight detection framework, HCFD-YOLOv8, was developed to address the challenges of small pest targets, scale variation, dense distributions, and complex background interference in pepper pest detection. Based on YOLOv8n, the proposed framework integrates CSP-HSA, CCFM, DyHead, and Inner-MPDIoU to collaboratively optimize feature extraction, cross-scale feature interaction, adaptive prediction, and bounding-box regression. This coordinated design enables HCFD-YOLOv8 to achieve an effective balance between detection performance and model efficiency.

Compared with YOLOv8n, HCFD-YOLOv8 improves Precision from 84.0% to 90.9%, Recall from 82.8% to 89.8%, mAP50 from 87.1% to 92.0%, and mAP50:95 from 54.3% to 55.2%. Meanwhile, the model reduces parameters from 3.00 M to 2.43 M and FLOPs from 7.5 G to 6.5 G. The framework achieves 39.40 FPS on the evaluated cloud-server platform, demonstrating competitive inference efficiency under the current experimental conditions. These results indicate that HCFD-YOLOv8 provides a favorable trade-off between detection accuracy and computational cost for pepper pest monitoring.

Nevertheless, the current study has several limitations. The proposed model was evaluated using a single constructed pepper pest dataset, and its performance on independent external datasets remains to be validated. Therefore, the reported results and conclusions should be interpreted within the scope of the dataset and experimental conditions evaluated in this study. The dataset also contains a limited number of pest categories and does not fully cover seasonal variations, extreme weather conditions, or diverse cultivation environments. Future work will focus on expanding the dataset and conducting external validation using independently collected datasets from different regions, seasons, cultivation environments, imaging devices, and pest populations. The model will also be validated on representative edge-computing platforms, and its runtime efficiency will be further optimized through model compression and deployment-oriented techniques. Additional evaluations involving memory consumption, energy usage, latency, and challenging environmental conditions will further assess the practical applicability and generalizability of the proposed framework.

## Figures and Tables

**Table 1:** Number of images and label counts for each category.

Dataset	Number of Images	Label Counts for Each Pest Category		
		Thrips	Caterpillars	Aphids
Train	2005	3854	743	5445
Val	250	446	251	639
Test	250	437	255	657
Total	2505	4737	1249	6741

**Table 2:** Functional roles and detection challenges addressed by the proposed modules in HCFD-YOLOv8.

Module	Position	Main Function	Detection Problem Addressed
CSP-HSA	Backbone	Enhance fine-grained feature extraction	Small pests and complex backgrounds
CCFM	Neck	Improve multi-scale feature fusion	Scale variation and occlusion
DyHead	Detection head	Adaptive feature prediction	Dense targets and appearance variation
Inner-MPDIoU	Loss function	Improve localization regression	Boundary uncertainty and overlapping targets

**Table 3:** Performance comparison of mainstream YOLO models.

Models	P/%	R/%	mAP50/%	mAP50:95/%	Pa/M	FLOPs/G
YOLOv5n	82.3 ± 0.23	80.0 ± 0.18	84.3 ± 0.13	49.5 ± 0.16	3.22	8.2
YOLOv8n	84.0 ± 0.31	82.8 ± 0.11	87.1 ± 0.41	54.3 ± 0.38	3.00	7.5
YOLOv11n	83.3 ± 0.11	82.2 ± 0.32	86.5 ± 0.15	53.7 ± 0.19	2.62	6.7

**Table 4:** Hardware and software configurations of the experimental platform.

Category	Item	Configuration
Computing platform	Platform type	Cloud server
Hardware	CPU	12-core vCPU, Intel(R) Xeon(R) Platinum 8352 V
Hardware	GPU	One 32 GB virtual GPU provided by the cloud computing platform
Hardware	System memory	90 GB
Software	Programming language	Python 3.9.7
Software	Deep learning framework	PyTorch 1.12.1
Software	GPU acceleration environment	CUDA 11.6
Model input	Input image resolution	640 × 640 pixels

**Table 5:** Ablation Study Results.

CSP-HSA	DyHead	CCFM	Inner-MPDIoU	P/%	R/%	mAP50/%	mAP50:95%	Pa/M	FLOPs/G
−	−	−	−	84.0 ± 0.31	82.8 ± 0.11	87.1 ± 0.41	54.3 ± 0.38	3.00	7.5
−	√	−	−	90.1 ± 0.06	88.5 ± 0.33	90.8 ± 0.13	54.9 ± 0.54	3.90	8.5
−	−	√	−	84.2 ± 0.11	83.4 ± 0.28	86.9 ± 0.27	53.1 ± 0.47	2.89	6.8
−	−	−	√	83.9 ± 0.17	83.9 ± 0.15	87.5 ± 0.33	54.8 ± 0.38	3.00	7.5
−	√	√	−	89.6 ± 0.24	88.6 ± 0.02	91.2 ± 0.15	54.3 ± 0.46	3.12	7.6
−	√	−	√	90.0 ± 0.35	88.2 ± 0.23	90.5 ± 0.32	55.1 ± 0.33	3.90	8.5
−	−	√	√	84.3 ± 0.19	83.1 ± 0.31	86.3 ± 0.40	54.8 ± 0.48	2.89	6.8
−	√	√	√	88.8 ± 0.20	87.5 ± 0.10	90.2 ± 0.17	54.5 ± 0.67	3.12	7.6
√	−	−	−	85.6 ± 0.32	84.6 ± 0.17	87.3 ± 0.21	53.9 ± 0.56	2.54	7.2
√	√	−	−	91.6 ± 0.08	90.4 ± 0.28	92.5 ± 0.14	56.3 ± 0.59	3.44	8.2
√	−	√	−	84.2 ± 0.22	83.5 ± 0.20	86.8 ± 0.18	51.3 ± 0.37	2.24	6.0
√	−	−	√	85.3 ± 0.11	84.3 ± 0.27	87.1 ± 0.29	53.9 ± 0.63	2.54	7.2
√	√	√	−	89.6 ± 0.14	84.5 ± 0.19	90.5 ± 0.11	53.4 ± 0.43	2.43	6.5
√	−	√	√	86.7 ± 0.31	85.4 ± 0.24	87.6 ± 0.13	52.6 ± 0.48	2.24	7.2
√	√	−	√	90.3 ± 0.16	88.4 ± 0.16	91.4 ± 0.34	53.8 ± 0.66	3.44	8.2
√	√	√	√	90.9 ± 0.04	89.8 ± 0.22	92.0 ± 0.10	55.2 ± 0.25	2.43	6.5

**Table 6:** Comparison of detection performance and computational efficiency among different object detectors.

Models	P/%	R/%	mAP50/%	mAP50:95/%	Pa/M	Weight Size/MB	FPS	FLOPs/G
YOLOv5n	82.3 ± 0.23	80.0 ± 0.18	84.3 ± 0.13	49.5 ± 0.16	3.22	6.53	48.21	8.2
YOLOv8n	84.0 ± 0.31	82.8 ± 0.11	87.1 ± 0.41	54.3 ± 0.38	3.00	5.97	43.14	7.5
Faster-R-CNN	51.3 ± 0.56	71.2 ± 0.47	71.1 ± 0.55	35.9 ± 0.47	66.7	255.40	6.62	256.8
RT-DETR	90.7 ± 0.14	87.2 ± 0.27	92.3 ± 0.20	55.0 ± 0.31	32.6	65.80	24.76	106.3
YOLOv11n	83.3 ± 0.11	82.2 ± 0.32	86.5 ± 0.15	53.7 ± 0.19	2.62	5.23	41.09	6.7
HCFD-YOLOv8	90.9 ± 0.04	89.8 ± 0.22	92.0 ± 0.10	55.2 ± 0.25	2.43	4.96	39.40	6.5

**Table 7:** Lightweight Module Comparison Results.

Backbone Network	P/%	R/%	mAP50/%	FLOPs/G	Model Size/MB
CSP-HSA	85.6 ± 0.32	84.6 ± 0.17	87.3 ± 0.21	7.2	5.0
CSPHet	83.9 ± 0.19	83.2 ± 0.45	87.6 ± 0.33	8.2	4.8
C2f-AKConv	82.4 ± 0.21	81.9 ± 0.11	86.1 ± 0.24	7.5	5.0
MobileNetV2	81.0 ± 0.22	81.6 ± 0.17	84.9 ± 0.22	8.6	5.0
ShuffleNetV1	82.8 ± 0.32	79.8 ± 0.16	84.6 ± 0.27	6.5	7.3
C2f-MSBlock	80.6 ± 0.33	79.0 ± 0.18	81.5 ± 0.11	7.9	6.1

## Data Availability

The complete implementation code and the dataset images that can be legally redistributed are publicly available at: https://github.com/1469820949-glitch/Repository-name-HCFD-yolov8 (accessed on 31 July 2026). The dataset annotation files and data split information are available from the corresponding author upon reasonable request. Some images used in this study were obtained from third-party public datasets and cannot be redistributed because of copyright restrictions. The original sources of these third-party images are provided in the manuscript, and the images may be accessed through the corresponding original platforms, subject to their terms of use.

## Appendix A. The Description of Symbols Is Explained in This Study

**Symbol**	**Description**
**Technical abbreviations**	
AP	Average Precision
BiFPN	Bidirectional Feature Pyramid Network
C2f	Cross-Stage Partial Feature-Fusion Module
CCFM	Cross-Scale Context Fusion Module
CIoU	Complete Intersection over Union
CNN	Convolutional Neural Network
CRFPN	Channel Recalibration Feature Pyramid Network
CSFF	Cross-Stage Feature Fusion
CSP	Cross-Stage Partial
CSP-HSA	Cross-Stage Partial Hybrid Spatial Attention
CUDA	Compute Unified Device Architecture
DConv	Deformable Convolution
DFL	Distribution Focal Loss
DIoU	Distance Intersection over Union
DWConv	Depthwise Separable Convolution
DyHead	Dynamic Head
DYReLU	Dynamic Rectified Linear Unit
ECA	Efficient Channel Attention
ELAN	Efficient Layer Aggregation Network
EMA	Efficient Multi-Scale Attention
F1-score	Harmonic mean of Precision and Recall
FLOPs	Floating-Point Operations
FN	False Negative
FP	False Positive
FPN	Feature Pyramid Network
FPS	Frames Per Second
GFLOPs	Giga Floating-Point Operations
GFL	Generalized Focal Loss
GIoU	Generalized Intersection over Union
GPU	Graphics Processing Unit
HCFD-YOLOv8	The proposed lightweight pepper-pest detection framework based on YOLOv8n
HSA	Hybrid Spatial Attention
HetConv	Heterogeneous Convolution
Inner-MPDIoU	Inner Multi-Perspective Distance Intersection over Union
IoU	Intersection over Union
mAP	Mean Average Precision
mAP50	Mean Average Precision at an IoU threshold of 0.5
mAP50:95	Mean Average Precision averaged over IoU thresholds from 0.5 to 0.95
MPDIoU	Multi-Perspective Distance Intersection over Union
NMS	Non-Maximum Suppression
P	Precision
PAN	Path Aggregation Network
PANet	Path Aggregation Network
Params	Number of trainable parameters
PR	Precision–Recall
R	Recall
R-CNN	Region-Based Convolutional Neural Network
ReLU	Rectified Linear Unit
RT-DETR	Real-Time Detection Transformer
SPP	Spatial Pyramid Pooling
SPPF	Spatial Pyramid Pooling Fast
TP	True Positive
ViT	Vision Transformer
YOLO	You Only Look Once
**Mathematical symbols and formula variables**	
*P*_3×3_	Number of learnable parameters in a standard 3 × 3 convolution layer
*P_Het_*	Number of learnable parameters in a HetConv layer
*C_in_*	Number of input channels
*C_out_*	Number of output channels
*G*	Number of convolution groups in HetConv; recommended replacement for P in Equation (2) to avoid conflict with Precision
*F_l_*	Fused feature map at feature-pyramid level l
*l*	Index of the current feature-pyramid level
*k*	Index of an input or neighboring feature-pyramid level in Equation (3)
*N*(*l*)	Set of neighboring feature-pyramid levels involved in fusion for level l
*α_k_^l^*	Dynamically learned weight assigned to the k-th feature map when generating the fused feature at level l
*DConv_k_*	Deformable convolution operation applied to the k-th feature map
*X^k^*	Input feature map at the k-th feature-pyramid level
*X*	Input feature map in the dynamic activation function
*Y*	Output feature map after dynamic activation
*a*_1_(*X*), *a*_2_(*X*)	Input-dependent scaling coefficients
*b*_1_(*X*), *b*_2_(*X*)	Input-dependent bias terms
*B_p_*	Predicted bounding box
*B_g_*	Ground-truth bounding box
*x_p_*, *y_p_*	Center coordinates of the predicted bounding box
*w_p_*, *h_p_*	Width and height of the predicted bounding box
*x_g_*, *y_g_*	Center coordinates of the ground-truth bounding box
*w_g_*, *h_g_*	Width and height of the ground-truth bounding box
*x*_1_*^p^*, *y*_1_*^p^*	Coordinates of the upper-left corner of the predicted bounding box
*x*_2_*^p^*, *y*_2_*^p^*	Coordinates of the lower-right corner of the predicted bounding box
*x*_1_*^g^*, *y*_1_*^g^*	Coordinates of the upper-left corner of the ground-truth bounding box
*x*_2_*^g^*, *y*_2_*^g^*	Coordinates of the lower-right corner of the ground-truth bounding box
|*B*|	Area of bounding box or geometric region B
*B_inner_*	Internal reference region defined by the intersection of the predicted and ground-truth bounding boxes
*IoU_inner_*	Intersection over Union calculated using the internal reference region
*d_b_^inner^*	Mean normalized internal boundary-distance discrepancy
*q* ∈ {*l*, *r*, *t*, *b*}	Boundary-direction index corresponding to left, right, top, and bottom; recommended replacement for k in Equation (11)
*δ_q_^p^*	Distance from the center of the predicted box to its boundary in direction q
*δ_q_^g^*	Distance from the center of the ground-truth box to its boundary in direction q
*ε*	Small positive constant used to prevent numerical instability or division by zero
*d_c_^inner^*	Normalized internal center-distance term
*w_inner_*	Width of the internal reference region
*h_inner_*	Height of the internal reference region
*L_Inner-MPDIoU_*	Final Inner-MPDIoU bounding-box regression loss
*λ_c_*	Weight coefficient of the internal center-distance term
*λ_b_*	Weight coefficient of the internal boundary-distance term
*λ_s_*	Weight coefficient of the internal shape-consistency term
*d_s_^inner^*	Internal shape-consistency penalty between the predicted and ground-truth bounding boxes
*F*_1_	F1-score, calculated as the harmonic mean of Precision and Recall
*i*	Object-category index in the mAP calculation
*K*	Total number of object categories; recommended replacement for k in Equation (16)
*P_i_*(*R_i_*)	Precision expressed as a function of Recall for the i-th category
*R_i_*	Recall variable for the i-th category
*N*	Total number of images processed during inference
*T*	Total processing time in seconds
